# Structure of O-Antigen and Hybrid Biosynthetic Locus in *Burkholderia cenocepacia* Clonal Variants Recovered from a Cystic Fibrosis Patient

**DOI:** 10.3389/fmicb.2017.01027

**Published:** 2017-06-08

**Authors:** A.A. Hassan, Rita F. Maldonado, Sandra C. dos Santos, Flaviana Di Lorenzo, Alba Silipo, Carla P. Coutinho, Vaughn S. Cooper, Antonio Molinaro, Miguel A. Valvano, Isabel Sá-Correia

**Affiliations:** ^1^Department of Bioengineering, Institute for Bioengineering and Biosciences, Instituto Superior Técnico, Universidade de LisboaLisboa, Portugal; ^2^Department of Chemical Sciences, University of Napoli Federico II Complesso Universitário Monte SantangeloNapoli, Italy; ^3^Department of Microbiology and Molecular Genetics, University of Pittsburgh School of Medicine, PittsburghPA, United States; ^4^The Wellcome-Wolfson Institute for Experimental Medicine, Queen’s University BelfastBelfast, United Kingdom

**Keywords:** *Burkholderia cepacia* complex, cystic fibrosis, chronic infection, O-antigen, lipopolysaccharide, clonal variation

## Abstract

*Burkholderia cenocepacia* is an opportunistic pathogen associated with chronic lung infections and increased risk of death in patients with cystic fibrosis (CF). In this work, we investigated the lipopolysaccharide (LPS) of clinical variants of *B. cenocepacia* that were collected from a CF patient over a period of 3.5 years, from the onset of infection until death by necrotizing pneumonia (cepacia syndrome). We report the chemical structure of the LPS molecule of various sequential isolates and the identification of a novel hybrid O-antigen (OAg) biosynthetic cluster. The OAg repeating unit of the LPS from IST439, the initial isolate, is a [→2)-β-D-Rib*f*-(1→4)-α-D-Gal*p*NAc-(1→] disaccharide, which was not previously described in *B. cenocepacia*. The IST439 OAg biosynthetic gene cluster contains 7 of 23 genes that are closely homologous to genes found in *B. multivorans*, another member of the *Burkholderia cepacia* complex. None of the subsequent isolates expressed OAg. Genomic sequencing of these isolates enabled the identification of mutations within the OAg cluster, but none of these mutations could be associated with the loss of OAg. This study provides support to the notion that OAg LPS modifications are an important factor in the adaptation of *B. cenocepacia* to chronic infection and that the heterogeneity of OAgs relates to variation within the OAg gene cluster, indicating that the gene cluster might have been assembled through multiple horizontal transmission events.

## Introduction

*Burkholderia cenocepacia* is a Gram-negative opportunistic human pathogen of the *Burkholderia cepacia* complex (Bcc), relevant in immunocompromised individuals and cystic fibrosis (CF) patients (Mahenthiralingam et al., 2002, 2005). *B. cenocepacia* lung infections in CF patients are associated with poor prognosis and increased risk of death (Mahenthiralingam et al., 2002, 2005; Drevinek and Mahenthiralingam, 2010). In comparison to *Pseudomonas aeruginosa*, the major CF pathogen, less is known about the molecular mechanisms involved in the adaptation of *B. cenocepacia* to the CF lung (Coutinho et al., 2011b; Maldonado et al., 2016).

The lipopolysaccharide (LPS) O-antigen (OAg) biosynthetic cluster is under strong selective pressure during chronic infection (reviewed in Maldonado et al., 2016). LPS is a major component of the Gram-negative bacterial outer membrane, which participates in host–bacterium interactions, such as adhesion, immune evasion, persistence, and antimicrobial resistance (Raetz and Whitfield, 2002; De Soyza et al., 2008; Valvano et al., 2011; Maldonado et al., 2016). LPS consists of a core oligosaccharide (core) that is covalently linked to a lipophilic glycan termed lipid A (Whitfield and Trent, 2014). In many bacteria, the LPS is extended by an OAg polysaccharide that is linked to the core. The lipid A is made of a β-(1→6)-glucosamine disaccharide acylated with primary fatty acids at positions 2 and 3 of both glucosamine residues, which are in turn phosphorylated at the 1- and 4′-positions. Secondary acyl chains can further substitute primary fatty acids at their hydroxyl positions (Whitfield and Trent, 2014). The core, subdivided into inner and outer core, comprises conserved monosaccharide residues, such as heptoses and 3-deoxy-D-manno-oct-2-ulosonic acid (Kdo), which are typically unique to the LPS molecule (Whitfield and Trent, 2014). In *Burkholderia* species, one of the Kdo residues is modified to D-*glycero*-D-*talo*-oct-2-ulosonic acid (Ko) (Silipo et al., 2005). The OAg extends away from the outer membrane surface becoming exposed to the extracellular milieu; it is composed of linear or branched homo- or heteropolysaccharides of variable length, with subunits consisting of up to eight different sugars (Valvano et al., 2011).

Recent studies at genome (Spencer et al., 2003; Lieberman et al., 2011; Traverse et al., 2013), transcriptome (Mira et al., 2011), and proteome (Madeira et al., 2011, 2013) levels indicate that the LPS and particularly the OAg undergo alterations during chronic infection, which could be attributed to bacterial adaptation to biofilm lifestyle (Lieberman et al., 2011; Traverse et al., 2013), immune evasion and selective pressure from antibiotics (reviewed in Maldonado et al., 2016). The airways of CF patients are colonized by polymicrobial communities that show variability in composition and diversity (Coburn et al., 2015). The CF microbiota is also exposed to a fluctuating environment and multiple selective pressures arising from nearly constant antibiotic treatment, oxygen limitation, and the persistent host inflammatory response (Döring et al., 2011; Cullen and McClean, 2015). Therefore, infecting bacteria give rise to genetically heterogeneous populations in which different phenotypes adaptive for growth and survival are selected (Harrison, 2007; Yang et al., 2011). Understanding the adaptive and evolutionary mechanisms within chronic infections of the CF airway may help improve the management of these infections.

We investigated a collection of serial clonal isolates of *B. cenocepacia* obtained from a CF patient over a period of 3.5-years since the onset of infection until the patient’s death with the cepacia syndrome (Cunha et al., 2003; Coutinho et al., 2011a). These isolates belong to the epidemic ET-12 lineage (represented by the prototypic strains J2315 and K56-2) (Mahenthiralingam et al., 2000; Holden et al., 2009). This patient was also co-infected with *B. multivorans*, another member of the Bcc, which appeared before the isolation of the early *B. cenocepacia* strain (isolate IST439); co-infection continued until near the patient’s death (Cunha et al., 2003). The *B. cenocepacia* isolates from this patient were characterized by phenotypic (Coutinho et al., 2011a), transcriptomic (Mira et al., 2011), proteomic (Madeira et al., 2011, 2013) and metabolic profiling (Moreira et al., 2016a), as well as by comparative genomics (still unpublished). Isolates of particular interest are *B. cenocepacia* IST4113 (a highly antibiotic resistant variant retrieved after an exacerbation treated with intravenous therapy), IST4134 (obtained just before the patient’s death), and IST4129 (a variant that exhibits attenuated virulence related with the loss of the third replicon (Moreira et al., 2016b). The virulence potential of these isolates in non-mammalian host models (Moreira et al., 2016b) and their ability to modulate dendritic cell function were also compared (Cabral et al., 2017).

In this study, we analyzed the chemical structure of the LPS molecule and the genetic organization of the predicted OAg biosynthetic cluster in these serial isolates. Our results reveal that the early *B. cenocepacia* IST439 isolate encodes a functional genetic cluster responsible for OAg biosynthesis, with a hybrid composition including genes highly homologous to *B. multivorans* genes. Further, this isolate produces a structurally different OAg from that previously reported in the ET12 lineage strains, while all subsequent *B. cenocepacia* isolates lost the ability to produce an OAg molecule.

## Materials and Methods

### Bacterial Strains and Growth Conditions

The isolates investigated in this study are indicated in **Table 1** (Cunha et al., 2003; Coutinho et al., 2011a). *B. cenocepacia* and *B. multivorans* isolates were recovered from the sputum of a CF patient at the major Portuguese CF Center in Hospital de Santa Maria (HSM), from Centro Hospitalar Lisboa Norte EPE, Lisbon, Portugal, between 1998 and 2002. These isolates belong to the same clonal complex and ET-12 lineage (Moreira et al., 2016b). Studies involving these isolates were approved by the ethics committee of the Hospital, and the anonymity of the patient was preserved. The genome of the soil strain *B. multivorans* ATCC 17616 (NCBI nucleotide accession number: NC_010804.1) (Vandamme et al., 1997) was used for comparisons. Bacterial cultures were stored at -80°C in 1:1 (v/v) glycerol. Bacteria were grown in LB Lennox (LB; Conda, Pronadisa) at 37°C with shaking at 250 rpm or in LB agar plates. *Escherichia coli* strains (**Table 1**) were grown in the same conditions. When needed, growth media were supplemented with antibiotics at the following concentrations: for *B. cenocepacia*, trimethoprim (TMP) at 100 μg/ml, and for *E. coli*, TMP at 50 μg/ml and kanamycin (KAN) at 40 μg/ml.

**Table 1:** Description of *Burkholderia* isolates and *E. coli* strains used in this study.

Bacterial isolate	Isolation date	Species	Description
IST419	2/26/1998	*B. multivorans*	Clinical isolates obtained from a chronically infected patient followed at the Cystic Fibrosis
IST439	1/30/1999	*B. cenocepacia* IIIA_ET-12	Center of Hospital Santa Maria, Lisbon, Portugal (Cunha et al., 2003;
IST4103	7/24/2001	*B. cenocepacia* IIIA_ET-12	Coutinho et al., 2011a; Moreira et al., 2016b)
IST4110	9/25/2001	*B. cenocepacia* IIIA_ET-12	
IST4112	10/11/2001	*B. cenocepacia* IIIA_ET-12	
IST4113	11/6/2001	*B. cenocepacia* IIIA_ET-12	
IST4116A	2/11/2002	*B. cenocepacia* IIIA_ET-12	
IST4116B	2/11/2002	*B. cenocepacia* IIIA_ET-12	
IST4131	2/26/2002	*B. cenocepacia* IIIA_ET-12	
IST4129	3/26/2002	*B. cenocepacia* IIIA_ET-12	
IST4130	5/14/2002	*B. cenocepacia* IIIA_ET-12	
IST4134	7/2/2002	*B. cenocepacia* IIIA_ET-12	
ATCC 17616		*B. multivorans*	Soil isolate (Berkeley, CA, United States) (Vandamme et al., 1997; Nishiyama et al., 2010)
DH5-α (pRK2013)		*E. coli*	Helper strain for triparental conjugation (Figurski and Helinski, 1979)
ER2925 (dam- dcm-)		*E. coli*	Host for plasmids used to transform resilient *Burkholderia* strains (Craig et al., 1989)

### LPS Extraction, Purification, and Compositional Analyses

Single-colony purified cells of the early isolate (IST439) and three late-stage clonal variants (IST4113, IST4129, and IST4134) were used for LPS purification and structural analysis. Bacteria were first grown overnight in LB broth until mid-exponential phase at 37°C with shaking (250 rpm). Cultures were diluted to an OD_640__nm_ of 0.2, and 100 μl of the cellular suspensions were plated onto LB agar plates and incubated for 24 h at 37°C. Bacteria were scraped from the agar surface, collected, autoclaved, and lyophilized. LPS from bacterial dried cells was extracted by the hot phenol/water method (Westphal and Jann, 1965). The nature of the extracted material was checked by SDS-PAGE after gel silver staining (Kittelberger and Hilbink, 1993). To remove contaminants the extracts were treated with RNase (Roth, Germany), DNase (Roth, Germany) and Proteinase K (Roth, Germany) at 37 and 56°C, followed by dialysis against distilled water. The LPS was further purified by ultracentrifugation (4°C, 30,000 rpm, 24 h) and gel-filtration chromatography. The monosaccharide content of the sample was determined by analysis of the acetylated O-methyl glycoside derivatives after treatment with HCl/MeOH (1.25 M, 85°C, 24 h) plus acetylation with acetic anhydride in pyridine (85°C, 30 min) using gas-liquid chromatography mass spectrometry (GLC-MS). The sugar linkages were determined as described (Ciucanu and Kerek, 1984). The total fatty acid content was determined on intact LPS by treatment with 4 M HCl (100°C, 4 h), followed by 5 M NaOH (100°C, 30 min). After extraction in chloroform, fatty acids were methylated with diazomethane and analyzed by GLC-MS. The ester bound fatty acids were released by base-catalyzed hydrolysis with aqueous NaOH 0.5 M, MeOH (1:1, v/v, 85°C, 2 h), and then the product was acidified, extracted in chloroform, methylated with diazomethane, and analyzed by GLC-MS. The absolute configuration of fatty acids was determined as previously described (Rietschel, 1976). Authentic 3-hydroxy fatty acids were used to assign the (R) configuration to all LPS/LOS acyl chains.

### NMR Spectroscopy

Prior to NMR spectroscopy an aliquot of the purified LPS (20 mg) was hydrolyzed with acetate buffer (100°C, 2 h). After centrifugation, the supernatant, containing the saccharide fraction, was collected, lyophilized, and purified by size exclusion chromatography. 1D and 2D ^1^H NMR spectra were recorded on a Bruker 600 DRX equipped with a cryo probe. The solvent employed was D_2_O and the temperature was 298 K and pD was 7. Spectra calibration was performed with internal acetone (δ_H_ 2.225 ppm, δ_C_ 31.45 ppm). The double-quantum filtered phase sensitive correlation spectroscopy (DQF-COSY) experiment was executed by using data sets of 4096 × 256 points. Total correlation spectroscopy (TOCSY) experiments were carried out with spinlock times of 100 ms, using data sets (t1 × t2) of 4096 × 256 points. Rotating frame Overhauser enhancement spectroscopy (ROESY) and Nuclear Overhauser enhancement spectroscopy (NOESY) experiments were performed by using data sets (t1 × t2) of 4096 × 256 points and by using mixing times between 100 and 400 ms. In all homonuclear experiments the data matrix was zero-filled in both dimensions to give a matrix of 4 K × 2 K points and was resolution enhanced in both dimensions by a cosine-bell function before Fourier Transformation. The determination of coupling constants was obtained by 2D phase sensitive DQF-COSY (Piantini et al., 1982; Rance et al., 1983). Heteronuclear single quantum coherence (^1^H, ^13^C HSQC) and heteronuclear multiple bound correlation (^1^H, ^13^C HMBC) experiments were recorded in ^1^H-detection mode by single-quantum coherence with protein decoupling in the ^13^C domain using data sets of 2048 × 256 points. ^1^H, ^13^C HSQC was performed using sensitivity improvement and in the phase-sensitive mode using Echo/Antiecho gradient selection, with multiplicity editing during selection step (States et al., 1982). The ^1^H, ^13^C HMBC experiment was optimized on long-range coupling constants with low-pass *J* filter to suppress one-bound connectivity, using gradient pulses for selection. A delay of 60 ms was employed for the evolution of long-range correlations. It was used a long-range coupling constant value of 6 Hz. The data matrix in both heteronuclear experiments was extended to 2048 × 1024 points using forward linear prediction (Stern et al., 2002).

### MALDI Mass Spectrometry

MALDI-TOF mass spectra of the intact LPS were recorded in reflectron mode and negative ion polarity on a Perseptive (Framingham, MA, United States) Voyager STR equipped with delayed extraction technology. Ions formed by a pulsed UV laser beam (nitrogen laser, λ = 337 nm) were accelerated by 24 kV. LPS/LOS preparation was executed as described before (Sturiale et al., 2005; Lombardi et al., 2013).

### Small-Scale LPS Extraction for SDS-Polyacrylamide Gel Electrophoresis Analysis

Lipopolysaccharide was extracted as previously described (Marolda et al., 1990) with small modifications. Briefly, *Burkholderia* isolates were harvested from overnight liquid cultures by centrifugation for 1 min after OD_640__nm_ adjustment to 2.0 in 1 ml of PBS, suspended in 150 μl of lysis buffer containing 2% SDS, 4% 2-β-mercaptoethanol, and 500 mM Tris-HCl (pH 6.8), and boiled for 10 min. Proteinase K (20 mg/ml) was added, and the sample was incubated at 60°C for 2 h. Finally, samples were mixed with the tracking dye solution (125 mM Tris-HCl [pH 6.8], 2% SDS, 20% [v/v] glycerol, 0.002% bromophenol blue, and 10% mercaptoethanol) and boiled for 5 min before the gels were loaded. LPS samples were resolved by electrophoresis (at 150 V for about 1 h 40 m) in 14% polyacrylamide gels with a Tricine-SDS system followed by silver staining as previous described to visualize the banding patterns of the OAg (Marolda et al., 2006).

### Genomic DNA Sequencing, Assembly, and Annotation

Bacterial cultures were prepared by suspending isolated colonies from LB agar plates in 3 mL LB broth, followed by overnight growth at 37°C with shaking at 250 rpm. Genomic DNA was extracted and purified using a DNeasy Blood and Tissue Lit (Qiagen, Germany) according to manufacturer instructions. DNA concentration and purity were assessed using a Nanodrop ND-1000 spectrophotometer.

*Burkholderia multivorans* IST419 was sequenced on an Illumina NextSeq500 at the University of Pittsburgh, Pittsburgh, PA, United States. Raw fastq paired-end files were processed for removal of Illumina adapters, trimming, and quality-based filtering using Trimmomatic (Bolger et al., 2014). *De novo* assembly was performed using Velvet with automated optimization of assembly parameters (Zerbino and Birney, 2008), using the two sets of pair-ended reads, and annotated using the prokaryotic genome annotation tool Prokka (Seemann, 2014). For the identification of mutational events in the OAg genetic clusters, read data sets were mapped against the relevant reference genomes (*B. cenocepacia* or *B. multivorans*) using BWA-MEM of BWA (Li and Durbin, 2010) and Bowtie 2 (Langmead and Salzberg, 2012), and visualized in IGV (Robinson et al., 2011).

DNA sequence reads for all isolates are available in the EMBL’s European Nucleotide Archive (ENA) under accession number PRJEB20052^1^). The sequence of the OAg cluster of *B. cenocepacia* IST439 is given as Supplementary Table S1.

### *In Silico* Characterization of O-Antigen Biosynthetic Gene Clusters

Genome sequences of 11 *B. cenocepacia* sequential isolates and the sequence of *B. multivorans* IST419 obtained from a CF patient (Coutinho et al., 2011a) were used to investigate the OAg biosynthetic cluster (the detailed analysis of the remaining genomic differences will be reported elsewhere). The gene composition of the OAg biosynthetic gene cluster was determined by examining the region flanked by genes *apaH* and *ureG* (Ortega et al., 2005). These flanking genes were detected by BLASTN (Altschul et al., 1990) using as queries the gene sequences from *B. cenocepacia* J2315 against each of the corresponding nucleotide databases created from the available sequences under study. The assembled scaffolds of all studied isolates were cropped to obtain only the OAg cluster (*apaH*-*ureG*) for further examination. Computer-assisted analysis for these clusters was performed in Artemis (Carver et al., 2008) and by BLASTP (Altschul et al., 1990) to reveal the open reading frames (ORFs). Then, the clusters were visualized and compared with the OAg cluster of *B. cenocepacia* K56-2 (NCBI nucleotide accession number: NZ_ALJA00000000.2) by Artemis and ACT (Carver et al., 2008), and gene function was assigned based on the analysis of predicted ORF homologies.

### Molecular Cloning of *wbiI* and *bmul_2510* Genes in *B. cenocepacia* Clonal Variants Lacking O-Antigen

Genes *wbiI* and *bmul_2510* were PCR amplified from *B. cenocepacia* IST439 chromosomal DNA using a Hot Start High Fidelity polymerase (Qiagen) and cloned into pSCrhaB2 (Cardona and Valvano, 2005; Cardona et al., 2006). Primers were designed based on the genome sequence of IST439 and included specific restriction enzyme sites and a sequence encoding the FLAG epitope tag (Supplementary Table S2). Primers WbiI–flag–NdeI and WbiI_439_XbaI were used to clone *wbiI* fused to an N-terminal FLAG tag; primers wbiI_439_NdeI and WbiI–flag–XbaI were used to clone *wbiI* fused to a C-terminal FLAG tag; primers Bmul_2510-439-NdeI and Bmul_2510-flag-XbaI were used to clone *bmul_2510* fused to a C-terminal FLAG tag; primers P1, P2, P3 and P4 were used to clone both *wbiI* and *bmul_2510* with C-terminal FLAGs using the Gibson Assembly strategy (New England BioLabs). The amplification conditions were 5 min at 95°C, 30 cycles of 95°C for 30 s, 60°C for 1 min, and 72°C for 2 min, and a final extension of 10 min at 72°C. The resulting products were digested with *Nde*I and *Xba*I, ligated to pSCrhaB2 and introduced into *E. coli* ER2925 (New England BioLabs) (Craig et al., 1989) by transformation. Transformants carrying recombinant plasmids with the DNA insert were screened by colony PCR with primers 824 and pSC rev, which anneal to vector sequences flanking the cloning sites. All constructs were confirmed by DNA sequencing.

### Plasmid Conjugation into *B. cenocepacia*

Plasmids were mobilized by triparental mating (Craig et al., 1989) into all *B. cenocepacia* clonal variants lacking the OAg that possess single nucleotide polymorphisms (SNP) in *Bmul_2510* and/or *wbiI* using *E. coli* DH5-α carrying the pRK2013 helper plasmid (Figurski and Helinski, 1979). Exconjugants were selected on LB agar plates supplemented with 100 μg/ml of TMP and 200 μg/ml of ampicillin (AMP).

### Western Blot

Protein expression in *B. cenocepacia* clonal variants expressing *wbiI* or *bmul_2510* from IST439 was confirmed by Western blot. Briefly, electrophoresed whole cell lysate samples were transferred to nitrocellulose membranes. The membranes were incubated with a 1:10,000 dilution of anti-FLAG mouse monoclonal antibody followed by incubation with a 1:5,000 dilution of a goat anti-mouse IgG monoclonal antibody conjugated to Alexa Fluor 680 (Life Technologies). Images were acquired using an Odyssey infrared imaging system (LI-COR Biosciences).

## Results

### Only the First of the 11 Sequential Clonal Variants of *B. cenocepacia* Produce OAg

**FIGURE 1:**
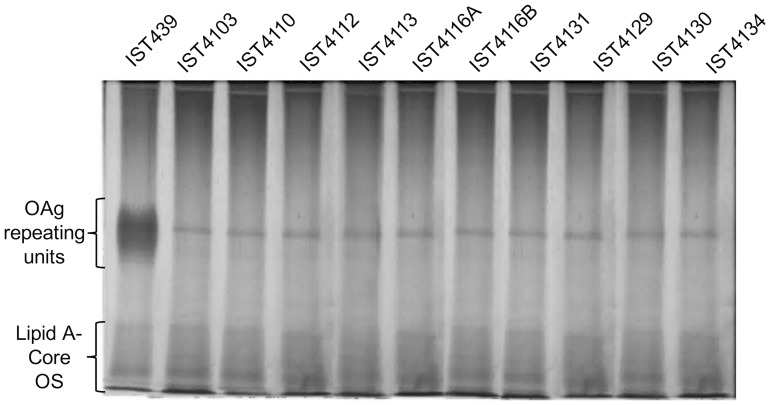
Electrophoretic profile of the LPS extracted from 11 clonal *Burkholderia cenocepacia* (*recA* lineage IIIA) isolates retrieved from a chronically infected CF patient (in chrononological order). LPS loading was standardized based on culture optical density. Samples were run in 14% polyacrylamide gels (at 150 V for about 1 h 40 m) in a Tricine-SDS system and developed by silver staining. The band that appears present in all the gels in the zone of the O-antigen (OAg) repeating units corresponds to residual proteinase K.

Chronically infecting bacteria in CF lungs often display changes in their LPS OAg (Maldonado et al., 2016). Therefore, we investigated the electrophoretic profiles of LPS extracted from the series of 11 sequential clonal variants of *B. cenocepacia*. SDS-PAGE followed by silver staining revealed that OAg was only present in the early isolate (IST439), while all other variants produce only lipid A-core devoid of OAg (**Figure 1**). The banding pattern of the OAg in IST439 was distinct from that in the strain K56-2 (Supplementary Figure S1). Therefore, although both IST439 and K56-2 belong to the same *B. cenocepacia* clonal group^2^ they produce seemingly different OAg molecules.

### Chemical Structure of the LPS of *B. cenocepacia* Isolates with or without O-Antigen

We investigated the chemical structure of the LPS produced by *B. cenocepacia* IST439 and also the structure of three randomly selected subsequent isolates that lacked OAg. Monosaccharide compositional analysis of the LPS from variants IST4113, IST4129 and IST4134 (**Table 1**) gave identical results (Supplementary Table S3), which also agreed with previously reported data for *B. cenocepacia* J2315 (Silipo et al., 2007). In contrast, the IST439 LPS had D-ribose and D-galactosamine residues as the main constituents. Results of linkage analyses of LPS from variants IST4113, IST4129, and IST4134 was also identical to that of J2315 (Silipo et al., 2007), but data obtained from IST439 LPS indicated also the presence of 2-substituted ribofuranose and 4-substituted galactosamine. The fatty acids content of the LPS in the four IST strains also matched the archetypal *Burkholderia* lipid A components, namely (*R*)-3-hydroxyhexadecanoic acid [C16:0 (3-OH)] in amide linkage, and (*R*)-3-hydroxytetradecanoic [C14:0 (3-OH)] and tetradecanoic acid (C14:0) in ester linkage (Silipo et al., 2007).

**FIGURE 2:**
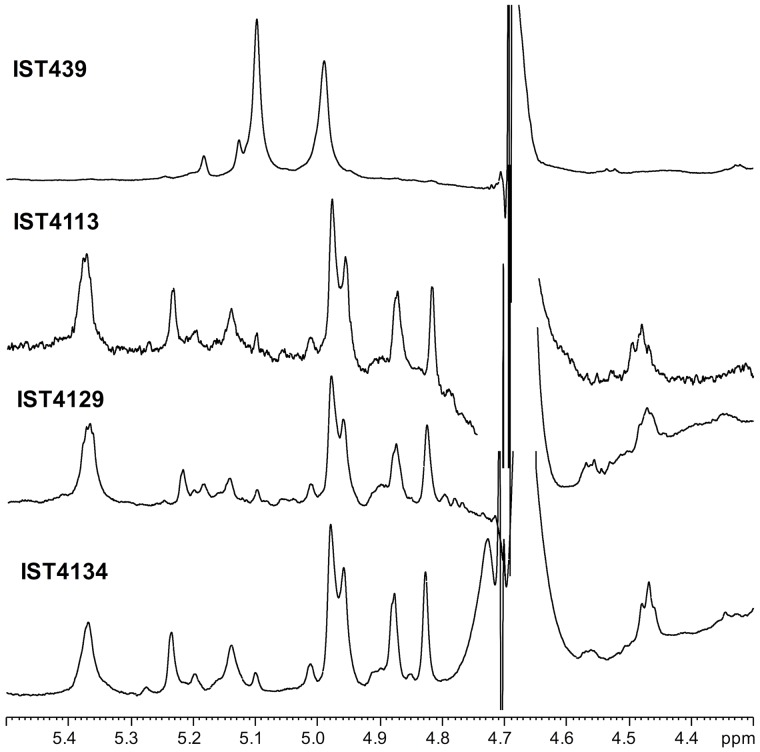
^1^H NMR spectrum of products obtained from IST439 and other three subsequent variants after mild acid hydrolysis.

**FIGURE 3:**
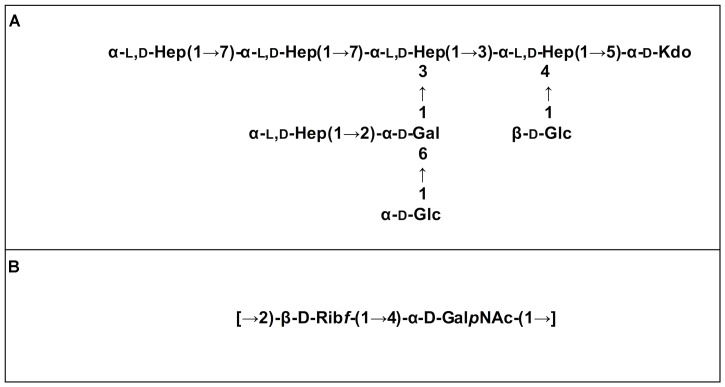
LPS moieties structures elucidated by NMR spectroscopy. Core structure of isolates IST439, IST4113, IST4129, and IST4134 **(A)** and OAg repeating disaccharide of isolate IST439 **(B)**.

Mild acidic conditions were used to characterize the monosaccharide sequence of the carbohydrate fraction of the purified LPS samples. This process cleaves the labile glycosidic linkage between the saccharide and the lipid A moieties. The carbohydrate fractions were purified by gel-permeation chromatography and analyzed by 1D and 2D NMR spectroscopy. The overlapped ^1^H-NMR spectra relative to all the isolates carbohydrate fractions were reported in **Figure 2**. It was immediately evident that the ^1^H NMR spectra of IST4113, IST4129, and IST4134 were identical, highlighting the presence of eight anomeric signals relative to the sugar units composing the core moieties. In contrast, the IST439 ^1^H NMR spectrum showed two main signals in the anomeric region (**Figure 2** and **Table 2**), attributed to the OAg domain. The complete LPS core oligosaccharide structures of isolates IST4113, IST4129, IST4134, and IST439 was assigned by the combination of data obtained from chemical analyses and 2D NMR spectroscopy using DQF-COSY, TOCSY, NOESY, ROESY,^1^H,^13^C-HSQC, and ^1^H,^13^C-HMBC. In all of these cases, the core was structurally identical to that previously described for *B. cenocepacia* J2315 (Silipo et al., 2007) except that the IST samples lacked the rhamnose-*N*-acetyl quinovosamine (Rha-QuiNAc) disaccharide linked to the outer core. In J2315, this disaccharide corresponds to a truncated component of the OAg (Ortega et al., 2009). **Figure 3A** shows the core structure, elucidated by NMR spectroscopy, of isolates IST4113, IST4129, IST4134.

**Table 2:** Chemical shift δ (^1^H/^13^C) of the O-chain moiety from *B. cenocepacia* IST439.

*Chemical shift* δ *(^1^H/^13^C)*					

**Unit**	**1**	**2**	**3**	**4**	**5**	**6**
A	5.09	4.15	4.03	3.95	3.77–3.59	–
2-β-Rib	106.6	78.7	67.2	82.3	62.5	–
B	4.99	4.02	3.93	3.61	3.63	4.03
4-α-GalNAc	95.5	50.0	76.2	78.9	60.5	70.8

**FIGURE 4:**
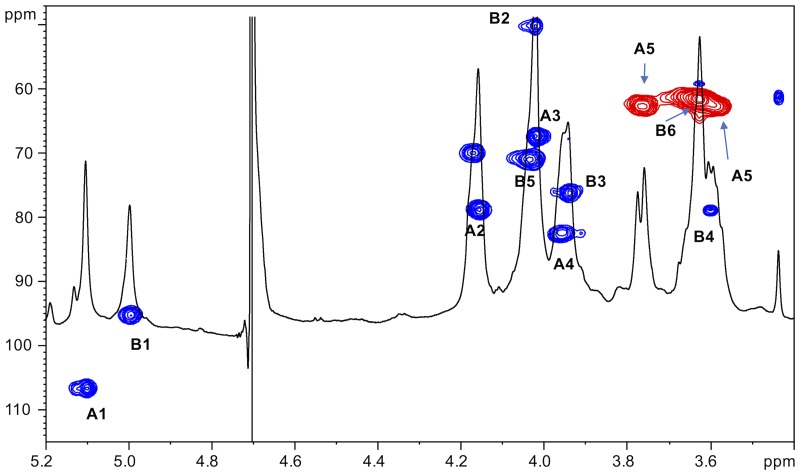
^1^H,^13^C-HSQC spectrum of the O-chain moiety from IST439 strain. The heteronuclear correlations are indicated.

As stated above, NMR spectroscopy of the carbohydrate fraction from isolate IST439 identified two spin systems in the ^1^H-NMR spectrum, belonging to the OAg, designed as **A** (H-1 at δ = 5.09 ppm) and **B** (δ = 4.99 ppm) (**Figure 2**). Spin system **A** was identified as ribofuranose due to the correlations observed in the DQF-COSY and TOCSY spectra from the H-1 **A** signal up to the diastereotopic methylene signal at position H-5. The furanose form was established due to the presence of the typical low field shifted ring carbon atom signals (**Figure 4** and **Table 2**). The anomeric configuration was attributed on the basis of the low ^3^*J*_H-1,H-2_ value attained from the DQF-COSY spectrum, indicative of a β-configuration in aldofuranose rings (less than 2 Hz), and by the chemical shift of C-4 (around 83.0 ppm in case of β-configuration) (Ahrazem et al., 2002, 2007). The downfield displacement of C-2 signal (**Figure 4**) indicated glycosylation at this position. Spin system **B** (H-1 at δ = 4.99 ppm) was identified as an α-*galacto*- configured monosaccharide, as attested by the low ^3^*J*_H3,H4_ and ^3^*J*_H4,H5_ values (3 and 1 Hz, respectively), and the TOCSY correlations between the anomeric signal and the other ring protons up to the H-4 resonance. Moreover, the anomeric proton and carbon chemical shifts (δ = 4.99 ppm and 95.5 ppm), the *intra*-residue NOE correlation of H-1 **B** with H-2 **B**, and the ^3^*J*_H1,H2_ were all in agreement with an α-anomeric configuration and a ^4^*C*_1_ ring conformation. The detection of a signal for C-2 at 50.0 ppm (**Figure 4**) indicated that C-2 was a nitrogen-bearing carbon atom. Moreover, the downfield shift of proton resonance of H-2 **B** was indicative of *N*-acetylation at this position, as also corroborated by the NOE contact of H-2 **B** with the methyl protons of the acetyl group resonating at δ = 1.92 ppm. Therefore, residue **B** was identified as a α-GalNAc. The downfield shift of its C-4 (δ = 78.9 ppm) was indicative of glycosylation at this position. ROESY and NOESY spectra (not shown) allowed to detect the dipolar correlations necessary to assign the primary sequence of the IST439 LPS OAg repeating unit. The anomeric proton of GalNAc **B** gave a NOE correlation with H-2 signal of ribose **A**, whose anomeric signal showed a NOE correlation with the H-4 of residue **B**. These results, confirmed by the long scalar range connectivity derived from the ^1^H,^13^C-HMBC spectrum (not shown), demonstrated that the IST439 OAg structure consisted of a repeating disaccharide of β-D-ribofuranose (β-D-Rib*f*) and α-D-*N*-acetylgalactosamine (α-D-Gal*p*NAc; **Figure 3B**). This structure is similar to that previously identified in *B. cepacia* (formerly *Pseudomonas cepacia*) serogroups O3 and O5 LPS OAg structures (Cox and Wilkinson, 1989).

**FIGURE 5:**
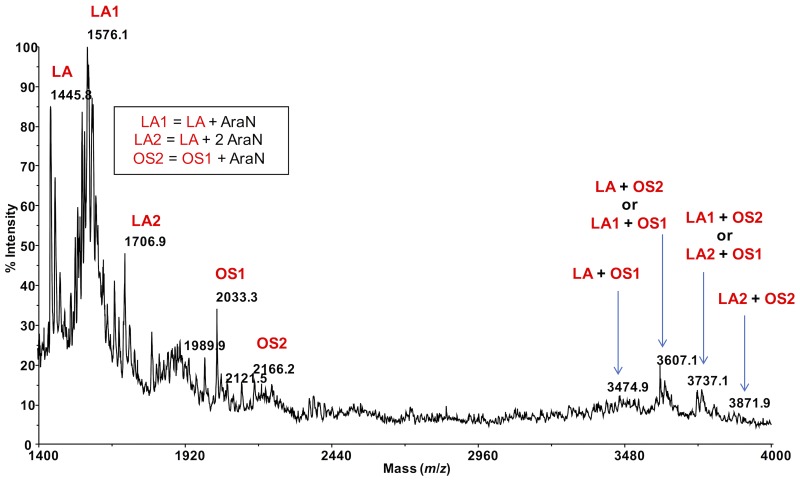
MALDI mass spectrum of the intact LPS from strain IST439 (mass range 1400–4000). Lipid A-core oligosaccharide molecular ions and their ion fragments, attributable to the core OS and to the reported lipid A structure(s), are indicated.

Negative-ion MALDI mass spectrometry (MS) of the intact IST439 LPS showed ion peaks corresponding to lipid A and core species, which arose from the cleavage of the labile glycosidic linkage between Kdo and lipid A. Ion peaks corresponding to lipid A species were assigned as previously described (Silipo et al., 2007). The ion peak **OS1** (*m/z* = 2033.3) agreed with an oligosaccharide composed of five heptoses, three hexoses, one Kdo, one D-*glycero*-D-*talo*-oct-2-ulosonic acid (Ko) and one 4-L-amino-4-deoxyarabinose (Ara4N), while ion peak **OS2** (*m/z* = 2166.2) corresponded to the same oligosaccharide carrying an additional Ara4N residue (**Figure 5**). Thus, the ion peaks derived from the IST439 core agreed with the structure previously elucidated (Silipo et al., 2007). The mass range 3000–4000 Da (**Figure 5**), containing peaks relative to either the oligosaccharide and the lipid A species, also confirmed previous studies (Silipo et al., 2007). Likewise, negative-ion MALDI mass spectra executed on the intact LPS isolated from the IST4113, IST4129, and IST4134 variants were identical each other and to the previously described mass spectra performed on intact *B. cenocepacia* ET-12 strain J2315 LPS (Ortega et al., 2009). The MALDI mass spectrum of the intact IST4113 LPS, presented in Supplementary Figure S2, showed at mass range *m*/*z* 1300–2500 Da, OS species composed of four to six heptoses and three to five hexoses, one Kdo, one Ko, and with or without Ara4N. In particular, the ion peak termed **OS** (*m/z* = 2032.5, Supplementary Figure S2) was indicative of the core previously elucidated (Silipo et al., 2007). The LPS molecular ions in the mass range 3000–4200 Da (Supplementary Figure S2) also confirmed previously reported data and agreed with chemical and NMR spectroscopy analyses.

### Genomic Sequence of the *B. cenocepacia* Clonal Isolates Reveals of a Novel Hybrid O-Antigen Biosynthetic Cluster

**FIGURE 6:**
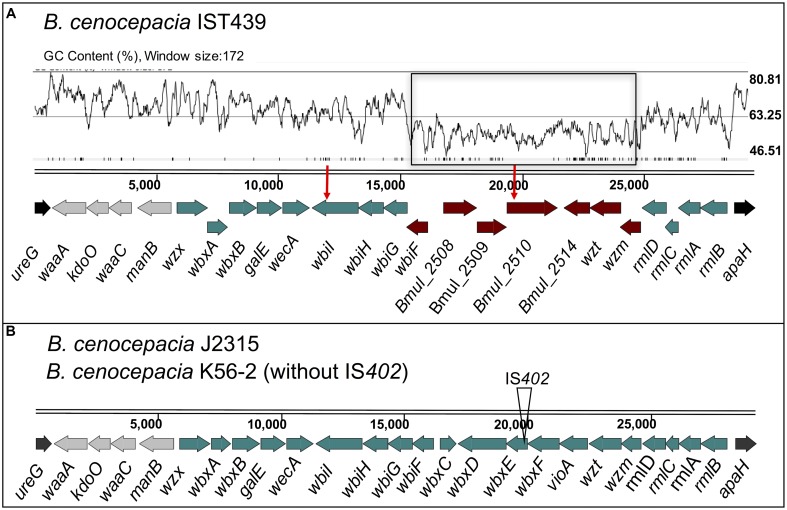
Genetic organization of gene clusters for core-lipid A and OAg biosynthesis in *B. cenocepacia* IST439 **(A)** and the reference strains K56-2 and J2315 **(B)**. The flanking genes *ureG* and *apaH* are indicated in black and the four genes represented in gray encode proteins putatively involved in lipid A-core biosynthesis. The genes in dark-red correspond to genes present in IST439 without a counterpart in the reference strain J2315 (Supplementary Figure S3), but with a degree of homology to *B. multivorans* ATCC 17616 (see also Supplementary Figure S4B). The red-vertical arrows above the genes represent the two non-synonymous point mutations in genes *bmul_2510* and *wbiI* of the 10 sequential isolates (comparative genomic analysis to be described elsewhere). A GC content plot is also represented for IST439 [drawn using Artemis (Carver et al., 2008)] above the display line of the sequence, where the genes annotated as *B. multivorans* are highlighted in a black rectangle. *wλ*, 3-deoxy-D-manno-octulosonic acid transferase; *kdoO*, Kdo dioxygenase; *waaC*, heptosyltransferase I; *manB*, phosphomannomutase; *wzx*, OAg exporter; *wbxA*, glycosyltransferase; *wbxB*, glycosyltransferase; *galE*, UDP-glucose epimerase; *wecA*, UDP-*N*-acetylglucosamine 1-P transferase; *wbiI*, nucleotide sugar epimerase-dehydratase; *wbiH*, UDP-*N*-acetylglucosamine 1-P transferase; *wbiG*, nucleotide sugar epimerase-dehydratase; *wbiF*, glycosyltransferase; *bmul_2508*, conserved hypothetical protein; *Bmul_2509*, group 1 glycosyl transferase; *Bmul_2510*, conserved hypothetical protein; *Bmul_2514*, type 11 methyltransferase; *wzt*, ABC transporter ATP-binding protein; *wzm*, ABC transporter membrane permease; *rmlDCAB*, dTDP-rhamnose biosynthesis; *wbxC*, acetyltransferase; *wbxD*, glycosyltransferase; *wbxE*, glycosyltransferase; *vioA*, nucleotide sugar aminotransferase.

**FIGURE 7:**
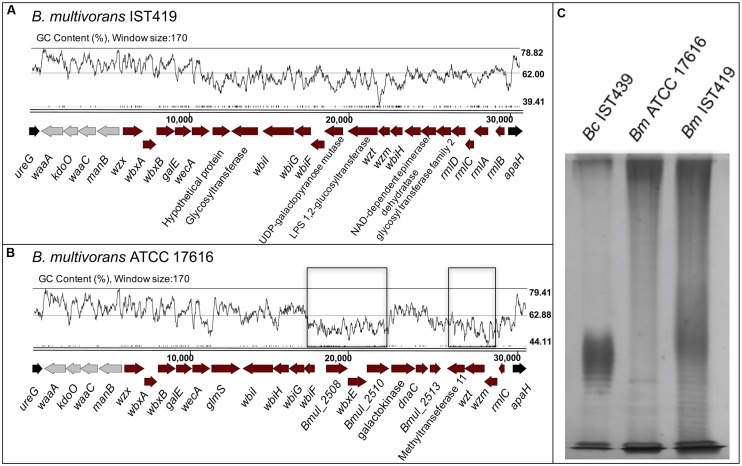
Genetic organization of the OAg biosynthetic gene clusters of *B. multivorans* strains ATCC 17616 **(B)** and IST419 **(A)**, including genes for lipid A-core biosynthesis and OAg biosynthesis within the *apaH* and *ureG* flanking genes (in black). Conserved genes among *Burkholderia* species involved in lipid A-core biosynthesis are indicated in gray. GC content plots are represented for both clusters [drawn using Artemis (Carver et al., 2008)], where the *B. multivorans* ATCC 17616 with homology in the corresponding cluster of *B. cenocepacia* IST439 are highlighted in a black rectangle. A silver nitrate-stained SDS-PAGE gel **(C)** shows the banding pattern of LPS samples extracted from *B. cenocepacia* IST439 (*Bc* IST439), *B. multivorans* IST419 (*Bm* IST419), and *B. multivorans* ATCC 17616 (*Bm* ATCC17616).

The 11 *B. cenocepacia* sequential isolates studied here belong to the *recA*-lineage IIIA/ET-12, as the prototypic epidemic strains K56-2 and J2315 (Coutinho et al., 2011a; Mira et al., 2011). The genome sequences of the 11 isolates were used to investigate the genetic organization of the OAg biosynthetic gene cluster in comparison to that of *B. cenocepacia* K56-2 (Ortega et al., 2005) (**Figure 6**). The *cenocepacia* K56-2 OAg biosynthetic cluster is on chromosome 1 and spans approximately 29 kb comprising 24 genes (**Figure 6**) flanked by *ureG* and *apaH* (Ortega et al., 2005). The *B. cenocepacia* IST439 OAg biosynthetic cluster is also located in chromosome 1, flanked by *ureG* and *apaH*, and spans a region of 29.5 kb. The cluster consists of 23 ORFs (**Figure 6**), most of them functionally assigned based on the bioinformatic analysis of predicted polypeptides. Like *B. cenocepacia* K56-2, the IST439 OAg cluster does not have a transposon insertion element inserted within the cluster (Supplementary Figure S3) (Ortega et al., 2005). Sixteen of the 23 ORFs in IST439 are homologs to *B. cenocepacia* K56-2/J2315 genes, including *wλ*, *wbxY* (*kdoO*), *waaC*, *manB*, *wzx*, *wbxA*, *wbxB*, *galE*, *wecA*, *wbiI*, *wbiH*, *wbiG, rmlD, rmlC, rmlA*, and *rmlB*. However, the remaining seven genes (*wbiF, bmul_2508, bmul_2509, bmul_2510, bmul_2514, wzt*, and *wzm*) are closer in terms of sequence similarity to ORFs in *B. multivorans* ATCC 17616 [environmental isolate with a complete genome deposited at NCBI and Bcc databases (Nishiyama et al., 2010)], ranging from 79 to 96% of amino acid identity, versus no homology (at an expect value cut-off of 1E-4) with any known *B. cenocepacia* strains for *bmul_2508, bmul_2509, bmul_2510*, and amino acid identity of 31 to 74% with the closely related ET-12 *B. cenocepacia* J2315 for the remaining four genes. This observation suggested a hybrid origin of this OAg cluster in IST439 (Supplementary Figures S2, S3). Analysis of the GC content across the IST439 OAg cluster shows a sharp drop in the region containing the seven genes related to *B. multivorans* (average content 56.1% in *B. cenocepacia* IST439 and 55.3% in *B. multivorans* ATCC 17616; **Figures 6A**, **7B**, respectively) in comparison to the rest of the cluster (average content 63.1%, and 66.6% when subtracting the seven genes) and the IST439 complete genome (average content 67.4%). This variation in GC content is usually found in genomic islands of prokaryotic genomes and is a hallmark of regions undergoing frequent recombination, as is the case of the LPS synthetic locus (Zhang et al., 2014; Maldonado et al., 2016).

The *wλ, kdoO, waaC*, and *manB* genes (represented in gray in **Figure 6**) encode proteins involved in lipid A-core biosynthesis (Ortega et al., 2005). In particular, the predicted amino acid sequence of Wλ and WaaC are highly identical to 3-deoxy-D-manno-octulosonic acid and heptosyltransferase I, respectively, and they are therefore involved in inner core LPS synthesis. *wbxY* (BCAL3311), previously annotated as a gene encoding a conserved protein of unknown function (Ortega et al., 2005), encodes a Kdo dioxygenase that is responsible for the conversion of the distal Kdo residue of the *Burkholderia* lipid A-core into Ko (Chung and Raetz, 2011; Chung et al., 2014; Fathy Mohamed et al., 2017), and it was renamed as *kdoO*. Another conserved functional feature in this OAg cluster is the predicted transmembrane protein encoded by *wbiH*, which includes functional features of the *wecA* family (Valvano, 2003; Ortega et al., 2005). The *wecA* family proteins and other proteins, encoded by *galE*, *wbiI*, and *wbiG*, are probably involved in the transfer of *N*-acetylhexosamines to the undecaprenol-phosphate intermediate to initiate the assembly of the OAg subunits (Raetz and Whitfield, 2002; Valvano, 2003, 2015), while genes *wbxA*, *wbxB, wbiF* and *bmul_2509* encode glycosyltransferases likely involved in OAg elongation. Genes *bmul_2508* and *bmul_2510* encode proteins of unknown function, while *bmul_2514* encodes a type-11 methyltransferase, possibly involved in the termination of OAg assembly. Genes *wzm* and *wzt* encode a two-component ABC transporter involved in OAg export across the cytoplasmic membrane (ABC-transporter-dependent pathway) after OAg polymerization on the cytoplasmic side (Raetz and Whitfield, 2002; Valvano, 2003, 2015). There is an alternative pathway of OAg export, the *wzy*-dependent pathway based on the concerted action of proteins Wzx and Wzy (Raetz and Whitfield, 2002; Valvano, 2003, 2015), but the OAg biosynthetic cluster of IST439 does not encode a homolog of *wzy*. This gene is also lacking in *B. cenocepacia* K56-2 (Ortega et al., 2005). Together, these observations indicate that the IST439 OAg, like in strain K56-2, is exported by an ABC transporter dependent pathway.

### Mutations in *wbiI* and *bmul_2510* Are Not Involved in Loss of OAg

**FIGURE 8:**
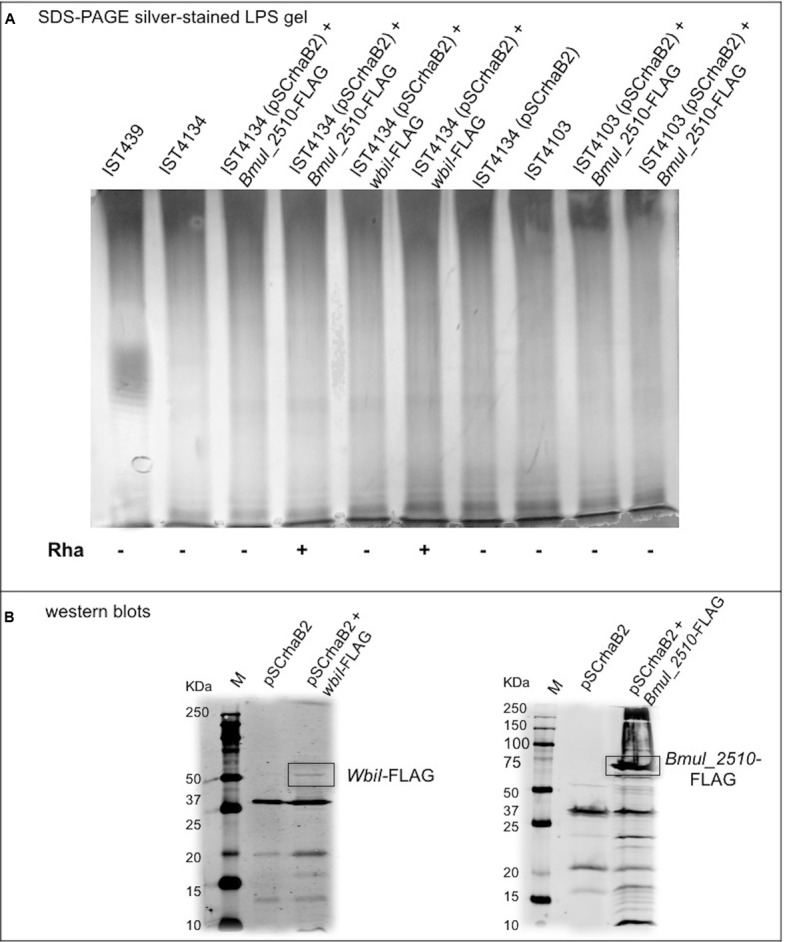
Characterization of the OAg unit after cloning and introduction of *B. cenocepacia* IST439 *wbiI* and *Bmul_2510* genes in all variants lacking OAg production to complement the corresponding mutated genotypes. **(A)** LPS electrophoretic profiles for IST439, IST4134, and IST4103 showing the presence/absence of OAg units, and OAg absence in all transconjugants selected; (+) denotes 1% (w/v) rhamnose and (–) denotes no rhamnose added to the medium. **(B)** Western blots showing bands at around 60 KDa (left) and 75 KDa (right), which indicate WbiI and Bmul_2510 polypeptides, respectively. Lanes shown (left to right): protein ladder (M), cloning vector pSCrhaB2, cloning vector + WbiI-FLAG or Bmul_2510-FLAG.

Whole-genome comparison of the 10 *B. cenocepacia* clonal variants against IST439 identified only three mutational events spanning the entire OAg cluster, corresponding to three non-synonymous SNPs in coding regions *IST439_01746* (homolog to *B. cenocepacia bmul_2510*) and *wbiI* (**Figure 6A**, red arrows). The point mutation in *bmul_2510* is conserved in all sequential isolates and results in the replacement of a threonine for a proline residue (T116P) in the corresponding Bmul_2510 polypeptide. In contrast, *wbiI* has two different point mutations resulting in L493P replacement in three isolates (IST4112, IST4113, and IST4116B) and E489K in other six isolates (IST4110, IST4116A, IST4131, IST4129, IST4130, and IST4134). No other mutations were identified within the OAg biosynthetic locus. Further, no mutations were found in the OAg ligase *waaL* and in the *wabO-dnaE* cluster that contains the reminder of the core-oligosaccharide biosynthesis genes (Ortega et al., 2009). However, eleven conserved SNPs were found outside the OAg cluster as well as one mutational event – as a deletion - in the late variants, compared with IST439, but none of them appear to be directly related to the loss of the OAg. Since the *bmul_2510* mutation is in all isolates after IST439, and *wbiI* mutations are present in nine isolates (except for IST4103), we performed complementation experiments in IST4103 with the *bmul_2510* homolog gene cloned from IST439 as an attempt to reconstitute OAg biosynthesis (Cardona and Valvano, 2005). The *bmul_2510* coding sequence including a FLAG tag epitope was placed under the control of a rhamnose-inducible promoter and the complemented strain was grown at various rhamnose concentrations. However, we could not detect OAg production in IST4103 (**Figure 8**). We also attempted complementation experiments for *bmul_2510* and *wbiI* singly or in combination using strain IST4134, but none of these experiments resulted in restoration of OAg synthesis (**Figure 8**). Failure to complement OAg synthesis was not due to lack of protein expression since *bmul_2510*- and *wbiI*-encoded polypeptides containing FLAG tags were detectable by Western blot analysis (**Figure 8B**). Additional bands reactive against the FLAG monoclonal antibodies correspond to cross-reactive proteins, as they also appear in the control lanes of lysates from bacteria containing empty vector. Therefore, from these results we conclude that the gene polymorphisms in the OAg biosynthesis cluster cannot explain the loss of OAg production in the sequential isolates obtained after IST439.

### Analysis of the OAg Cluster of a Co-infecting *B. multivorans* Isolate Rules Out that the Hybrid Cluster in IST439 Arose from Gene Transfer in the Lung

Because of the hybrid nature of the IST439 OAg biosynthetic cluster and the isolation of co-infecting *B. multivorans* in the same patient, we investigated the possibility that this cluster could have arisen from a gene transfer event in patient. *B. multivorans* IST419 was collected approximately 1 year before *B. cenocepacia* IST439 in patient J (**Table 1**) (Cunha et al., 2003). The combination of two sequencing rounds of *B. multivorans* IST419 allowed us to assemble 155 contigs (N50 = 171331) with a predicted genome size of ∼6.5 Mb and 5818 genes (Zerbino and Birney, 2008; Seemann, 2014). Using this annotated assembly, the corresponding OAg genetic cluster was identified and compared to that of *B. multivorans* ATCC 17616 (Nishiyama et al., 2010), *B. multiv*orans D2095 (NCBI nucleotide accession number: JFHP00000000.1) (Silva et al., 2016), and *B. cenocepacia* IST439. The OAg biosynthetic cluster in *B. multivorans* IST419, also located in chromosome 1, includes 25 ORFs spanning a 30.8 kb segment flanked by *ureG* and *apaH* (**Figure 7A**). Functional assignments could be made to most of the genes based on bioinformatics. This OAg cluster has more ORFs than IST439 but shows three conserved features: first, the region involved in lipid A-core biosynthesis (*wλ*, *kdoO*, *waaC*, and *manB*), second, the presence of *wbiH* and *wecA* encoding the putative initiating enzyme for OAg biosynthesis, and third, the presence of conserved genes *rmlBACD* encoding enzymes for dTDP-rhamnose synthesis (Valvano, 2003, 2015; Ortega et al., 2005). A detailed analysis of this cluster, together with the alignment of filtered fastq files of *B. multivorans* IST419 against the same region of *B. multivorans* ATCC 17616 (**Figure 7B** and Supplementary Figure S4C), shows that *B. multivorans*-like genes in the IST439 OAg cluster are not present in *B. multivorans* IST419 (Supplementary Figure S4A). Further, a similar analysis using a second clinical *B. multivorans* isolate as reference (*B. multivorans* D2095 (Silva et al., 2016)), also revealed the complete absence of homologs to those specific *B. multivorans* ATCC 17616 genes. The electrophoretic analysis of LPS samples obtained from *B. cenocepacia* IST439, *B. multivorans* IST419, and *B. multivorans* ATCC 17616 showed that the first two express an OAg possessing repetitive units with different banding patterns, but *B. multivorans* ATCC 17616 does not produce an OAg unit (**Figure 7C**). Therefore, we conclude that *in vivo* gene transfer over the course of the infection of the patient can’t explain the hybrid gene organization of the OAg cluster in IST439 and related serial isolates.

To shed more light on the origin of the hybrid OAg biosynthetic cluster in *B. cenocepacia* IST439, we determined the genetic organization of the corresponding region in the environmental isolate *B. multivorans* ATCC 17616 (**Figure 7B**). The gene cluster spans a region of 31.4 kb in chromosome 1 containing 24 predicted ORFs. In terms of functional conservation, the *B. multivorans* ATCC 17616 region is similarly organized to that of *B. cenocepacia* IST439 (Supplementary Figure S4B) although, unlike *B. cenocepacia* IST439, the seven genes annotated in *B. multivorans* ATCC 17616 are not consecutively organized in the OAg cluster (indicated by black boxes in **Figures 6A**, **7B**).

## Discussion

Phenotypic diversity and genotypic flexibility of *B. cenocepacia* during long-term infection of CF lungs was recently well established using genomic and phenotypic analyses of serially collected clinical isolates (Lee et al., 2017). In this study, we characterized a novel hybrid genetic LPS OAg locus in the early *B. cenocepacia* isolate of a series of sequential isolates obtained over a 3.5-year period from a patient with CF. We also demonstrate that all of the 10-subsequent clonal *B. cenocepacia* variants collected from the same patient until shortly before death do not produce OAg (**Figure 1**). This observation provides further evidence for the OAg loss during *B. cenocepacia* adaptation over chronic lung infection (Maldonado et al., 2016).

We elucidated the chemical structure of the lipid A-core of four of the 11 isolates, as well as the structure of the OAg in the early isolate (IST439). The lipid A-core of these strains had the same structure as that described for other members of the *Burkholderia* genus (De Soyza et al., 2004, 2008). Indeed, the lipid A moiety shows the typical *Burkholderia* glucosamine disaccharide backbone with the [P→4-β-D-Glc*p*N-(1→6)-α-D-Glc*p*N1→P] sequence that comprises two Ara4N residues linked to phosphate groups. The inner core contains the characteristic Ara4N-Ko-Kdo trisaccharide, previously elucidated in other *Burkholderia* species (Isshiki et al., 1998; Gronow et al., 2003), and is extended by a heptose-rich sequence. The OAg repeating unit of the LPS in IST439 was a disaccharide [→2)-β-D-Rib*f*-(1→4)-α-D-Gal*p*NAc-(1→], which was not previously described in *B. cenocepacia*, differs from the OAg structure of the prototypic strain K56-2 (Ortega et al., 2005, 2009), and is similar to that occurring in the OAg of *B. cepacia* serotypes O3 and O5 (Cox and Wilkinson, 1989). In addition to the presence of a novel OAg, the absence of the Rha-QuiNAc disaccharide, linked to the α-Hep-(1→2)-α-Gal disaccharide of the outer core in all the isolates investigated, represented a further novelty of these strains.

In keeping with the OAg structure, a new OAg biosynthetic locus was also discovered in this study. This genetic locus exhibits a hybrid origin, comprising genes with homology to *B. multivorans* ATCC 17616 and low to no amino acid similarity with other sequenced *B. cenocepacia* strains. Comparisons with the OAg locus of a co-infecting *B. multivorans* isolate (IST419), which was present at the time of acquisition of *B. cenocepacia* IST439 (**Figure 7A**) (Cunha et al., 2003), showed that the genetic organization of the OAg cluster in *B. multivorans* IST419 is different from that of *B. cenocepacia* IST439, with no homology with the *B. multivorans*-like genes shared by IST439 and environmental strain *B. multivorans* ATCC 17616 genomes. Together, our findings demonstrate that no transfer of genetic material occurred between the two co-infecting species in the lung of this CF patient during the infection within the OAg biosynthetic cluster.

The origin of the unique OAg locus of *B. cenocepacia* IST439 is unknown, but it was found that the *B. multivorans*-like region differs significantly from the GC content of the entire cluster and core genome (**Figure 6A**), which is a common indicator for the presence of a putative genetic island and former mobile element (Hacker and Carniel, 2001; Zhang et al., 2014). Studies mostly based on *Salmonella enterica* and *E. coli* (D’Souza et al., 2005; Hu et al., 2010; Reeves et al., 2013) have previously suggested that OAg biosynthetic operons in bacterial pathogens were acquired by horizontal genetic transfer from species with a low GC content. Moreover, horizontal exchange of O-specific antigen biosynthetic genes among phylogenetically distinct *P. aeruginosa* strains was observed and serotype switching was found to be the result of horizontal transfer and genetic recombination of LPS biosynthetic genes originating from an multidrug-resistant taxonomic outlier *P. aeruginosa* strain (Thrane et al., 2015). The high variation within the cluster also indicates that these OAg clusters might have been assembled by multiple transmission events over time (Lerouge and Vanderleyden, 2002; Thrane et al., 2015; Maldonado et al., 2016). In the case of *B. cenocepacia* IST439, the core genome and the OAg cluster (minus the 7-gene region) have a similar GC content (66–67%), whereas the region containing the seven genes annotated as *B. multivorans* ATCC 17616, with a 56.1% GC content, is an obvious candidate for a mobile element that was acquired by horizontal genetic transfer and may be adaptive. However, the corresponding seven genes in *B. multivorans* ATCC 17616 are not encoded consecutively, being interrupted by a group of three high-GC content genes (**Figure 7B**), and cannot be matched to the same region in *B. cenocepacia* IST439 without assuming the occurrence of multiple mutational events. Based on these observations and the variability observed within the OAg cluster of the *B. multivorans* sequences examined, it can be suggested that this putative mobile element identified in the OAg biosynthetic cluster of *B. cenocepacia* IST439 has a foreign non-Bcc origin, which at some point might have also been acquired by an ancestor of *B. multivorans* ATCC 17616.

We could not establish a direct link between OAg loss in all 10 late-stage variants with the conserved mutation in the gene homolog to *Bmul_2510* or with the mutations in *wbiI* since none of our complementation efforts using genetic constructions with *B. cenocepacia* IST439 *Bmul_2510* and/or *wbiI* in different backgrounds could reconstitute OAg biosynthesis. Although it is possible that lack of complementation of OAg synthesis is associated with insufficient expression of the cloned gene or poor translocation of the protein to the membrane, it cannot be excluded that these mutations require the complementation of another genetic alteration in a distinct part of the genome with some role in OAg biosynthesis that remains to be identified. Alterations in the LPS molecule during chronic CF infections are thought to contribute to adhesion, evasion of immune defenses and overall adaptation to the infection niche (Maldonado et al., 2016). In *P. aeruginosa*, it is well established that adaptation to the lung during chronic infection includes the loss of OAg and/or lipid A modifications that allow the bacterium to avoid host immune responses (Lyczak et al., 2002; Cigana et al., 2009; Dettman et al., 2013; Maldonado et al., 2016). LPS virulence resides both in the endotoxin activities of the lipid A and in the ability of the core oligosaccharide and OAg to provide the bacterium with resistance to host defenses. The ability of 3 of the 11 *B. cenocepacia* clonal variants to subvert host defenses was recently assessed using dendritic cells (Cabral et al., 2017), revealing that the late variants IST4113 and IST4134 were significantly more internalized than IST439, the only isolate that expresses the OAg unit, in line with previous studies by Saldias et al. (2009). Moreover, the late-stage isolates also exhibited improved survival within dendritic cells than the early isolate IST439, corroborating the idea that loss of the OAg may participate in providing an adaptive advantage to chronically infecting *B. cenocepacia*. Collectively, our results lend support to the notion that LPS OAg modifications are an important factor in the adaptation of *B. cenocepacia* to chronic infection and that that OAgs heterogeneity relates to variation within the OAg gene cluster.

## Author Contributions

FDL, AS, and AM were responsible for LPS-O-Ag structural analysis and CC and IS-C prepared Bcc cells for the analysis. RM was involved in the cloning and expression of O-Ag genes in MV laboratory and AH, CC, and RM performed the electrophoretic profiles of LPS and the complementation assays. SdS, VC, and IS-C were involved in the genome sequencing and analysis of the *B. cenocepacia* clonal isolates and AH, SdS, RM, MV, VC, and IS-C in the *in silico* analysis of Bcc O-Ag clusters. IS-C coordinated the work and the writing of the paper with contributions from the co-authors. All authors have read and approved the submission of this manuscript.

## Conflict of Interest Statement

The authors declare that the research was conducted in the absence of any commercial or financial relationships that could be construed as a potential conflict of interest.

## Footnotes

1 http://www.ebi.ac.uk/ena/data/view/PRJEB20052

2 http://pubmlst.org/bcc
