# Supplementary material for: Structure of O-Antigen and Hybrid Biosynthetic Locus in Burkholderia cenocepacia Clonal Variants Recovered from a Cystic Fibrosis Patient
Source: Front Microbiol. 2017 Jun 8;8:1027. doi: 10.3389/fmicb.2017.01027 (PMC5462993; doi:10.3389/fmicb.2017.01027)
Supplement: Supplementary file 1 [file Table_1.pdf]

Supplementary Material

Structure of O-antigen and hybrid biosynthetic locus in *Burkholderia cenocepacia* clonal variants recovered from a cystic fibrosis patient

A. Amir Hassan[1§], Rita F. Maldonado[1§], Sandra C. dos Santos[1§], Flaviana Di Lorenzo[2§], Alba Silipo[2], Carla P. Coutinho[1], Vaughn S. Cooper[4], Antonio Molinaro[2], Miguel Valvano[3] and Isabel Sá-Correia[1*]

*** Correspondence:** Professor Isabel Sá-Correia: isacorreia@tecnico.ulisboa.pt

§ These authors contributed equally to this work

1 Supplementary Tables:

Table S1: The sequence of the OAg cluster of *B. cenocepacia* IST439

>IST439_01731 3-deoxy-D-manno-octulosonic-acid transferase
ATGCTGAGGGCGATCTATCGCGCGCTGTGGTGGCTCGTCGCGCCGGCCGCGGTCATCCGG
CTCTACGTGCGCTCGCGCAAGGAGCGCGGTTATCGCGAGCATATCGGCGAGCGCTTCGGT
CATGTCGCGGGCCGCTCGCGCGACGACCGCGCGCCGCTGATCTGGGTGCATGCGGTGTCG
GTCGGCGAGACGCGCGCCGCGCAGCCGCTGATCGATGCGCTGATGCGCGCGCGCCCCGAT
GCGCGCATCCTGCTCACGCACATGACGCCGAGCGGCCGCGCGACCGGCGAACAGATCTTC
GGCGATCGCGTGCTGCGCTGCTACCTGCCGTACGACATGCCGGGCGCGGTGCGGCGCTTC
CTGCGTGCATGGCGGCCGACGCTCGGCCTCGTGATGGAAACCGAGGTGTGGCCGACGCTG
ATCGACGAGTGTCGCCGCGCGGACGTGCCGCTGGTGTTGACCAATGCGCGGATGTCCGCG
CGTTCGTTCCGGCGCGCGGCGAAGTTCGGCGCGGCGACGCGCGACGTGTTCGGCGGCTTC
TCGCGCGTGCTCGCGCAGAGCCCGGCCGATGCGGAGCGGCTGACGTCGCTCGGCGCCCGC
AACGTGACCGTGCTCGGCAACCTGAAGTTCGACATGACGACGCCGCCGGAACTCGCGGCA
CGCGGCCATGCGTGGCGCGACGCGATCGGCGCGCGGCCGGTGTGGGTCGCCGCCAGCACG
CGCGAGAACGAAGAGGCGCTGGTGTTGCAGGCGTTCGCGGAAATGCGTACGCCCGGCGCG
CTGCTGGTGCTCGTGCCGCGTCATCCGCAGCGCTTCGCCGAGGTCGAGGCGCTCGTCGCG
CGCGGCGGGCTCAAGTGCGTGCGGCGCTCGGTGTGGGCGGCCGATGCGGCCGCGCTCGCA
GCGGGCCGGCCGGCGGCCGACCCGCTGCCGGACGACGTCACGGTGTTGCTCGGCGATTCG
ATGGGCGAGCTCGGCGCGTACTACGCGGCGGCCGACATCGCGTTCATCGGCGGCAGCCTG
CTGCCGCTCGGCGGGCAGAACCTGATCGAAGCGTGCGCGGTCGGCGTGCCGGTGCTGATC
GGGCCGCACGTGTTCAACTTCACGCAGGCGACCGCCGATGCGGTCGCGGCCGGTGCGGCG
ATGCAGGTCGAGGATCCGCTCGATCTCGCACACGTGCTCGACGCGCTGTTCGCCGACAAC
GCGCGGCGCATCGCGATGGGCGCGGCCGGCGCGGCCTTCGCATCGCGTCACCGCGGCGCG
ACCGCGCGCACGGTCGACGTGCTCGCCGCGCTGTTGCCGCCTGCCGCAACCGGCGCGCGT
GCGCTGCCGGACGTGCAGGACGCTCCGGGCGACGACGCATAG
>IST439_01732 hypothetical protein
ATGAGCGAATCCCAGATCATCGAAGTCCCGTCCGCCGACTGGAGCGGACACAACCTGTCA
GCGCCGCGCGAGCAGTTGCTGGCCGCGGTCGAGGAAGGCAAGGTGCTGTATTTCCCGCAC
CTGCGCTTCGCGATCGAAGGCGGCGAGGAAGCGCTGCTCGATCCGGCGCTCGCCGATCCG

AAACGCAAGAACATCAGCCTCGCGCCGAACGGCGGCGCTCGCCGGCGTGCTCGGCGAC
AGCGTCACGCAGTCGGCCGTGCGCGCGCTCGTCGCGCGCTTCCAGCAGCAGGCCGGCACG
CTCGTCGACGGCCTCTTTCCCGAATACCGCGGCAAGCTGCGCGTCGCGCCGACGAGCCTG
CGGCTGATGCAGGTCGAAACGCGCCAGACGTCGTGGCGCAAGGACGACAGCCGGCTGCAC
GTCGACGCGTTCCCGTCGCGGCCGAACTACGGCGAGCGCATCCTGCGCGTGTTCACGAAC
GTGAACCCGGCCGGCGCGCCGCGCGTGTGGCGTGTCGGCGAGCCGTTCGAGGACGTCGCG
AAGCGCTTCCTGCCGAAGATCCGGCCGCAGTTCCCGGGCTCGGCGTGGCTGCTGAACCTG
CTGCACGTGACGAAATCGCCGCGCAGCGCATACGACCATCTGATGCTGAACCTGCACGAC
GGGATGAAGGCCGACCTCGACTACCAGAAGACGTGTCCGCAGCAAACGATGCCGTTTCCG
CCGGGCAGCGTGTGGATATGTTTCTCCGATCAGACTTCGCACGCTGTGATGTCGGGCCAG
TTCATGCTCGAGCAGACCTTCTTCCTGCCGGTCGACGCGATGGTTCGCCGCGAATGCGCG
CCGCTCGGCATTCTCGAACGCCTGACGGGCAGGGCGCTGGTTTGA
>IST439_01733 lipopolysaccharide heptosyltransferase I
GTGCAAAAGATCCTGATCGTGCGTGTGTCGTCGCTGGGCGACGTCGTGCACAACATGCCG
GTGATCGCCGATATCCGGCGCCGCCACCCCGATGCGCAGATCGACTGGCTCGTCGAGGAA
AGCTTCGTCGACCTCGTACGGCTCGTCGACGGCGTGCGCAACGTGCTGCCGTTCTCGCTG
CGCCGCTGGCGCAAGAAGCCGTTCTCGGGGGCCACGTGGCGCGAGATCCGCGCGTTCCGC
CGGCGCCTCGCGGCCGAGCAGTACGACCTCGTGATCGACTGCCAGGGCCTCATCAAGACC
GCGTGGGTCGCGAGCTGGGCCCGCGGCCCGCTCGTCGGGCTCGGCAACCGCACCGACGGC
GCCGGCTACGAGTGGCCGGTGCGCTTCTTCTACCGCAAGCGCGTGCCGATCGCGCCGCGC
ACGCACGTCGTCGAGCGCTCGCGCCAGTTGGTCGCGGCCGCGCTGGACGACCCGGCGCCA
ACCCCGGCCGACCCCGTCGAGTTCGGCCTCGACACGCGGGCGGCAGCGCTCGCGGTGGCC
GCGCTCGGCCTGAACTTGCCGGTGCCGTACGTCGTGTTCGTCCACGCGACGTCGCGCGCC
GACAAGCAGTGGCCGGACGCCGCGTGGATCGAGCTCGGCCAGGCGCTGGTGCGGCGCGGC
GCGTCGCTCGTGCTGCCGTGGGGCAACGACGCGGAACGCGCGACCAGCGAGCGGCTCGCG
AAGGAATTCGGTGCGGCGGCGATCGTGCCGCCGAAGCTGTCGCTGCCGGCCGTGGTCGGG
CTGATCGACGGCGCGGCCGCGACGGTCGGGGTTGATACAGGTCTGGTTCACATCGCGGCC
GCGCTGAAGCGCCCGACGGTCGAACTGTACAATTTCGCGACGGCCTGGCGGACCGGCGGC
TACTGGTCGCCGAACGTCGTCAATCTCGGCACGGCGGGGCAGCCCCCGTCGATCGCGCAG
GTGAAGTCGGCGCTCGCGGGCTTCGGCCTCCTGTAA
>IST439_01734 phosphomannomutase
ATGATCTCCCAATCCATCTTCAAGGCATATGACATTCGTGGCGTGGTCGGCAAGACCCTC
GACGTCGACACGGCGCGCGGGATCGGCCGGGCATTCGGCAGCGAAGTGCGTGCGCAGGGC
GGCGATGCGGTCGTCGTCGCGCGCGACGGCCGCCTGTCCGGACCGGAGCTGGTCGGCGCG

CTGGCCGACGGCCTGCGTGCGGCCGGGGTCGACGTCGTCGACGTCGGCATGGTGCCGACG
CCGGTCGGTTATTTCGCGGCGAACGTGCCGCTCGCGCTGAAGGGCGGCGAGCGCCGCGTC
GATTCGTGCATCGTCGTCACGGGCAGCCACAACCCGCCCGACTACAACGGCTTCAAGATG
GTGCTGCGCGGCGCCGCAATCTACGGCGAGCAGATCCAGGCGCTGTACCGCCGCATCGTC
GACGAGCGGTTCGAGACGGGCAGCGGCACGTATGAAGCAATCGACGTCGCGGATCAGTAC
ATCGCGCGCATCGTCGGCGACGTGAAGCTCGCGCGGCCGCTGAAGCTCGTCGTCGACGCC
GGCAACGGCGTCGCGGGCCCGCTCGCGACGCGCCTGTTCAAGGCGCTCGGCTGCGAACTC
GTCGAGCGCTTCACCGACATCGACGGCACGTTCCCGAATCACCACCCCGATCCCGCCCAC
CCGGAAAACCTGCAGGACGTGATCCAGGCGCTGAAGGACACCGATGCGGAGCTCGGCTTC
GCGTTCGACGGCGACGGCGACCGTCTCGGCGTCGTCACGAAGGACGGCCAGATCATCTAT
CCGGACCGCCAGCTGATGCTGTTCGCGGAAGAAGTGCTGTCGCGCAATCCGGGCGCCCAG
ATCATCTACGACGTGAAGTGCACGCGCCACCTCGCGCAGTGGGTGAAGTCGAAGGGCGGC
GAGCCGCTGATGTGGAAGACCGGCCATTCGCTCGTGAAGGCGAAGCTGCGCGAGACGGGG
GCACCGCTGGCCGGCGAAATGAGCGGCCACGTGTTCTTCAAGGATCGCTGGTACGGCTTC
GACGACGGCCTGTACACGGGCGCGCGCCTGCTGGAAATTCTCGCGAAGACGGCCGATCCG
AGCGCGCTGCTCAACAGCCTGCCGGACGCGATGAGCACGCCGGAACTGCAGCTCTGGCTC
GACGAAGGCGAGAATTTCCGCCTGATCGAGCAGTTGCAGAAGGAAGCGAAGTTCGACGGC
GCCGACGAAGTCGTGACGATCGACGGCCTGCGTGTCGAGTACCCGGACGGCTTCGGCCTC
GCGCGTTCGTCGAACACGACGCCGGTCGTCGTGATGCGCTTCGAAGCCGAGACGCAGGAA
GGGCTCAAGCGCATCCAGGAGGACTTCCGCCGCGTGCTGACGGCCGCGAAGCCGGACGTC
AAGCTGCCGTTCTGA
>IST439_01735 membrane protein
ATGCTGAAGCGCTTCGGCAACCCGGACGTCGCGAAGGCCGTCGCGAACCTCGTCTGGCTG
GGGCTCGAACGGCTCACCCAGATCGGCGTCGCGATCGCGATCAGCGGCCTGCTGGCCCGT
TATTTCGGGCCAGATGTGTTCGGCAAATGGCAGTATGCGAATACGCTTCTCCTGGTACTG
GCGCCGCTCACCTGGGTGTGCGGCGCGGAAATCCTCGTCCCGACCATCGTCCAGCGCCCG
CCCGCGCAGCTCGGCGCGGTGCTCGGCAGCGCGTTCGTGCTGCGCATCGCCGTGTCGGCC
GCCGCGCTCGCCGCGACCTGGATCGCGATCGCCGCGGGCGCCTTCGACCCGCTGGTCGGC
GCGATGCTCGCGGGCCTCGCGGTCACGATGGTGTTCCGCGAGCCGTTCGTCGGCGTGATC
AACGCGTGGCTGCAGAGCATGACCTACAGCAAGCCGCAGCTCGTCACCAGCATGGTCACC
GCGCTCGCCAAGGCGCTGCTCGTCTGGCTGCTGGTCCGCGCGGCCGCCGGCCCCGCCCGC
TTCGCGTGGCTGTGGGCGCTGGAAGCCGCCGCGATCGGCTTCGCGCTGCTGCTGTACTAC
CGCCATCGTAACGGCGGTGCGCTCGGCTGGACGTTCGACAAGCCGCTGTTCCGGCACTTT
GCAACGGCCGGCACCGTGTTCTGGCTCGGCCTCATCTGCATGTACCTGTTCCTGAAGCTC

GACCGCCTGATGCTCGAGCGCCACGTGTCGTTCGCCGATCTCGGCCGCTATTCGGCCGCG
CAGCAGCTCAACGAGAACTGGATCACGCTCGCGCTGATGCTCGCGCAGACGATCGCGCCC
GCCTTCGTCTACCGCGTGCAGGACGTCGCGCGGCTGCGCCGCAACATCGTCCGGCTGATC
GCGATGACGGCCGGCCTGATGACGGCCGGCGCGCTCGTGCTCGACGCCGCCGCCCCGCTG
ATCGTCGGCAAGGTGTTCGGCCGCGGCTACGAGGCGTCGGTCGACATCTTCCGCTGGGCC
GTCTGGCTGTCCGTGCCGGCCGGCATCGAGGCGATAGGCAATCTTATCGTTCTCAAATAT
CAAGCAAAATTCGTGTTGCTGTCGAAATGGCTGCTCGCGCTGGCCATCGCCGCGATCGTC
AACCTGTTCGCGATCCCGCGGCTCGGCCTGTACGGCGCGCTCGTCGGGCTGGCGGCCGGC
TACCTGGCCGCGGCCGCCGTCAACTTTTATTACATCCGTTTCAAGCTGCGAACATGA
>IST439_01736 glycosyl transferase family protein
ATGACGTCCCCTGCTTGCCCGACCCCGCTCGACGACGTGGCCGTGCTGATGCCGGCCTAC
AACGGGCACGACGACGTCGTCCGGACCCTCGCGTCGTTTCGCGAGGACGCGCCCGTGCAC
GTGCTGATCGTCGACGACGGCAGCACGCCGCCGATCGTCGCGCCCGATCTGCCGGGCCTG
TCGATCGACGTGCTGCGCATGCCCCGGAACGGCGGCATCGAACGCGCGCTGGCGGCCGGC
ATCGACGCGCTCGCGGCGCGCGGCTTCCGCTACGCGGCCCGTATCGACGCCGGCGATCTC
GCCGCGCCGCAGCGCCTCGCGAAGGAGCGCGCCTATCTCGGCGCCCACCCGCGCGTAGCC
TGCGTCGGCATGTGGACGCAGGTCGTGTCGCGCGCCGGCGAGCCGCGCTTCATGCTGACG
CCGGCCGCCGATCCGCGCACGCTGCGCCGCACGCGCTTCCTGCGCTCGCCGCTCGTGCAT
CCGTCTGTGATGTTGCGCATCGACGCCGTGCGCGAAGTGGGCAACTATCGGGCAAAGTAC
CGCGCGGCCGAGGATCTCGATCTTTTTTTACGGTTAATGCAACGCTACGATTGCGCGAAC
CTGCCGGAACTCGGCCTGTATTACGAGCTTAACGAGGGCGGGATCAGCGCGACCAAACGC
CGGCGCCAGCTGGTGTCGACGCTCACGCTGCTGCTGCGCCACTTCAACGTGCTGAACCCG
TACGACTGGGCCGGCCTCGCCAAGAACCTGCTGCATTTCGTGACGCCGTACCGTACGCTG
CAGCGCATCAAGCAGACGCTGTTCGCCGCGCGGCCGTCCGCCTGA
>IST439_01737 group 1 glycosyl transferase
ATGTCCGCCACCTCCCCGCTGCGCATCGCGCTCGTCTGCAACACGGCCTGGGCGATCCAT
ACGTATCGCCACGGGCTGATCCGTGCGCTCGTCGCGCGCGGCGCCGAGGTCGTCGTGATC
GCGCCGCACGATCGCACCGTGCCGTTGCTCGAGCAGATGGGCTGCCGCTACGTGGCGCTC
GCGGTCGCGTCGAAAGGCACGAGCCCGCGCGAGGATCTCGGCACGCTCGCCGCGCTGGTG
CGCCACTATCGCGCGCTGCGGCCCGACCTCGTGTTCCATTACACGATCAAGCCGAACATC
TACGGCTCGGTGGCCGCGTGGCTCGCGCGCGTGCCGTCGATCGCGGTGACGACGGGCCTC
GGCTACGTGTTCATCCAGAAGAGCCGCGCGGCGAGCGTCGCCAAGCGCCTGTACCGGTTC
GCGTTCCGCTTCCCGCGTGAAGTGTGGTTCCTGAACCGCGACGATCTCGCGACCTTCACC
GACGAGCGCTTGCTCGCGCACCCCGATCGCGCACGGCTGCTGCACGGCGAAGGCGTCGAT

CTCGAACAGTTCGCGCCCGTGCCGCTGCCCGCCGGCGACGCGCCCGTGTTCATCCTGATC
GGCCGGCTGCTGTGGGACAAGGGCGTGCGCGAATACGTGGAGGCCGCGCGTCTCGTGCGC
GAGCGCTTCCCGAACGCGCGCTTCCAGTTGCTCGGGCCGCTCGGCGTCGACAATCCGAGC
GCGATCGGCCGCGCGGATGTCGACGCGTGGGTCGACGAAGGCATCGTCGAATATCTCGGC
GAAGCGCACGACGTGCGACCGCACATCGCGGCGGCCGACTGCGTCGTGCTGCCGTCGTAC
CGCGAAGGCGTGCCGCGCACGCTGATGGAAGCATCGGCGATGGGCCGCCCGATCGTCGCG
ACCGACGTGCCGGGCTGCCGCGACGTCGTCGCCGACGGCGAAACAGGTTACCTGTGCCGT
GTGCGCGACAGCGCGAGCCTCGCGGAGCAATTGACTCGCATGATCGAACTCGGAACGGCC
GGACGCGAAGCGATGGGCGCGCGCGGCCGGCAGAAGGTCACGGCGGAATTCGACGAACAG
CAGGTCGTCGAACGCTACCGGCGTACCATCCACTCGTTAACCGGTATCACCCTCTGA
>IST439_01738 UDP-glucose 4-epimerase
ATGACCGCTAAAGGCACCATCCTCGTCACCGGCGGAGCCGGCTATATCGGCTCGCACACG
ACCGTCGAGCTGCTCGACAACGGCTACGACGTCGTGATCGTCGACAACCTCGTCAACAGC
AAGGCCGAATCGGTGCGCCGGATCGAGCGGATCACGGGCAAGACGCCCGCGTTCCACCAG
GTCGACGTGTGCGACGAAGCCGCGCTCGCGAAGGTGTTCGACGCGCATCCGATCACCGGC
ACGATTCACTTCGCGGCGCTCAAGGCCGTCGGCGAATCGGTCGAGAAGCCGCTCGAGTAC
TACCAGAACAACATCGGCGGGCTGCTCGCCGTGCTCAAGGTCATGCGCGAGCGCAACGTC
AGGCAGTTCGTGTTCAGCTCGTCCGCGACCGTGTACGGCGTGCCCGAGCGCTCGCCGATC
GACGAATCGTTCCCGCTGTCCGCGACGAACCCGTACGGCCAGTCGAAGCTGATCGCCGAA
CAGATTCTGCGCGATCTCGAAGTCTCGGACCCGTCGTGGCGGATCGCGACGCTGCGCTAC
TTCAACCCGGTCGGCGCGCACGCGAGCGGGCTGATCGGCGAGGATCCGGCCGGCATCCCG
AACAACCTGATGCCGTATGTCGCGCAAGTCGCGGTCGGCAAGCTGGAAAAGCTGCGCGTG
TTCGGCTCGGACTACCCGACGCCGGACGGCACCGGCGTGCGCGACTACATCCACGTCGTC
GATCTCGCGAAGGGGCACATCGCCGCACTCGACGCGCTCGCGAAGCGCGACGCGAGCTTC
GTCGTGAACCTCGGCACCGGGCAAGGCTACAGCGTGCTCGAAGTGGTGCGCGCGTTCGAG
AAGGCGTCGGGTCGTCCGGTGCCGTACGAACTCGTCGCGCGCCGCCCGGGCGACATCGCC
GAGTGCTATGCGAACCCGCAGGCCGCGGCCGACATCATCGGCTGGCGCGCGACGCTCGGC
ATCGAGGAAATGTGTGCCGACCACTGGCGATGGCAGGAGGGGAACCCGCGCGGTTTTGTA
TAA
>IST439_01739 glycosyl transferase family protein
ATGCTCAGCTTCGCGTCCGGCTTCATCGTCTCCCTGCTCGTCACGCTGTTCATCGTGCGC
TACGCGCACCTTCACGAGAAATTCTCGATCGACAGCGACCTGGCCGGCGTGCAGAAATTC
CATGTGCGGCCGGTGCCGCGGGTGGGCGGCATCGGGATCCTCGCCGGGGTCGTCGTTGCC
GCGCTGGTGCTGTTCCGGCGCTATCCGACGATCTCCGGCAGCATTCTCGGGATCGTTGCC

TGCGGGCTGCCGGCGTTCCTGTCCGGCCTCGTCGAGGATCTGACCAAGCGCGTATCGCCG
CGTGCGCGGCTGCTCTGCACGATGGGCGCGGCCGCGCTCGCATTCTGGCTGATGCACATC
GCGGTCACGCGCATCAGCGTGCCGCCGCTCGACTTCCTGCTCGGCTACGTCGCCATTTCG
GCGTTCGTCACCGTGCTCGCGGTCGCCGCGCTCGCGAATGCGATCAACATCATCGACGGC
TTCAACGGGCTCGCGTCGATGGTCAGCTTCATGATGTTCGCGTCGCTCGCGTATGTCGCG
TTCCACGTCAACGACCCGGTCGTGATGTCCGCGTCGATCATCATGATGGGCGCCGTGCTC
GGCTTCTTCCTGTGGAATTTCCCGGCCGGGCTGATCTTCCTCGGCGACGGCGGCGCGTAT
TTCATCGGCTTCATGCTCGCGGAACTCGCGATCATGCTCGTGATGCGCAACCGCGAAGTG
TCCGCGTGGTACCCGGTGCTGCTGTTCATGTATCCGATCTTCGAGACCTGCTTCTCGATC
TACCGGAAGAAGTTCATTCGCGGGATGTCGCCAGGGATTCCGGACGGCGTGCACCTGCAC
ATGCTCGTGTACAAGCGGCTGATGCGCTGGGCCGTCGGCACGAAGCATGCGCACGACCTG
ACGCGGCGCAATTCGCTGACTTCACCTTATCTGTGGCTGCTGTGCCTCGTCGCGGTGATA
CCGGCGACGCTGTTCTGGCGGCATACGGTGCACCTGTTCGCGTTCGTCGTGCTGTTCGCG
GTGACTTATGTGTGGCTGTACATCAGTATCGTGCGGTTCCGCGCACCGCGATGGATGGTC
GTACGCAAGCATCGGCACAGCCGCTGA
>IST439_01740 polysaccharide biosynthesis protein CapD
ATGTTGCAATCCAGAGCATCTTGGCTGTCCCTGAGTGCTTTCCTGTTCGACCTGACGGCG
GTTGTCGCCGCATGGTTGTTCGCCTATCTCGTTCGTTTCAATGGCAGCGTTCCGCATGAT
TTCCTGAGCGGCGCGCTGATGGCGCTGACGTGGGTACTGCCCGTCTACGCGCTGATGTTC
CACGGGTTCGGCCTATATCGCGGGCTGTGGGTGTTCGCGAGCCTGCCGGACCTAATGCGT
ATCGCGAAAGCGGTGACCGGCGGCGGCATGATCGTGATGATCGGCGCCGTGATGTTCCAG
CCGGCACCGATCATCCCCCGCTCAGTGCTGCTCGTGTCGCCGCTGATGCTGTTCCTGGCG
ATGGGTGGCGCGCGCGCGCTGTATCGCGCGACGAAGGAGTACTACCTGTACGGCGGACTC
GTCGGCAAGGGCAAACCGGTGCTGGTGCTCGGCGCCGGCACGGCCGGCGCGAGCCTGGCA
CGCGAACTGTCGCGCTCCGGTGAATGGCGCCTCGTGGGCCTGCTCGACGACGACGTCACG
AAGCAGGGCCGTGAAATCTACGGCTACAAGGTGCTCGGCTCGTTCAACGACCTCAAGCAC
TGGACCGATGCGATGAAGGTCGAATACGCGATCATCGCGATTCCGTCGGCATCGGTCGAA
ACGCAGCGCCGTGTCGCGACCCTGTGCGTGCGGGCCGGCGTGAAGGCGATGGTGCTGCCT
TCGCTGACCGCGCTGATGCCGGGGCAGGGCTTCCTGTCGCAGGTACGGAACATCGATCTC
GAGGATCTGCTCGGCCGCGAGGCCGTGACGATCGATACGCCCCACGTCGAGGCGCTGCTG
CGCGGCCGCGTCGTGATGGTGACGGGCGCGGGCGGCTCGATCGGCTCCGAGCTGTGCCGG
CAGATCTTGCGCTTCGCGCCTGCACAGCTCGTTGCGTTCGACCTCTCGGAATACGCGATG
TACCGGCTGACCGAGGAGTTGCGCGAGCGGTTTCCGGATTTGCCCGTGGTGCCGATCATC
GGAGACGCGAAAGATTCGCTGCTGCTCGATCAGGTGATGTCGCGCCATGCGCCGCACATC

GTGTTCCATGCGGCCGCCTACAAGCACGTGCCGTTGATGGAAGAGCACAACGCATGGCAG
GCGCTGCGCAACAACGTGCTCGGCACCTATCGCGTGGCGCGGGCGGCGATCCGCCACGGC
GTGCGTCACTTCGTGCTGATCTCGACCGACAAGGCGGTCAATCCGACCAACGTGATGGGC
GCGAGCAAGCGTCTCGCCGAAATGGCCTGTCAGGCGTTACAGCAGACGAGCGGGCGCACC
CAGTTCGAGACGGTGCGCTTCGGCAACGTGCTCGGCAGCGCGGGCAGCGTGATTCCGAAG
TTTCAGCAGCAGATCGCGAAGGGCGGCCCGGTCACGGTCACGCACCCGGAGATCACGCGT
TTCTTCATGACGATCCCGGAAGCGTCGCAGCTCGTGCTGCAGGCGTCGAGCATGGGGCAT
GGCGGCGAGATCTTCATTCTCGACATGGGCGAGCCGGTGAAGATCGTCGATCTCGCGTGC
GACCTGATCCGCCTGTACGGTTTCTCGGAAGAACAGATCCGGATCGAATTCACCGGGCTG
CGGCCGGGCGAGAAGCTTTACGAGGAACTGCTCGCGGACGACGAGGCGACGACGCGCACG
CCGCACCCGAAACTGCGGATCGCGCGGGCGCGCGAAGTGCCGGACAATTTCCTCGACGAA
CTGCTGCCGTGGCTGATGCAGCATCGTGTGCTGCCCGACGACGAAGTGCGGCGCGACCTG
CGGCGCTGGGTGCCTGAGTACCAGACGGCCACCGTGCCAGCCCTGAAGAGTGTGAGAGTC
GCATCGAACCGCTGA
>IST439_01741 glycosyl transferase family protein
ATGCATTTCCCGATCTCCACTTGGCCTGCCGCGGTCGGGGTGGCGCTGGTCGCCGCCATC
GCATCGACGGCCATACTGCGCATGCTGCTCGCGACCGGCCTCGCATGGCGGCTCGCTACC
GATATTCCGAACGACCGTTCGCTCCATACGTTGCCGACGCCGCGTGTCGGCGGATGGGGC
ATCGTGCCAGTGTCCGTTGCCACGCTGTTGATGCTGGCACCGCAACTGTGGCTGGTCGCA
GTCGCAGCAGCAGGGCTGGCCGCGATGTCGCAGATTGACGACCGGCGCGGGCTGCCGGCG
CGCGTGCGGTTCTCGGCGCACCTCGCGGCAGTCGTTGCGCTGATCGTTGTATTTCCAGCC
GACGCGCCCTGGTGGCTGCTCGCTGGCGTGGGTTTCGCGATGGTCTGGCTGACGAACCTG
TACAACTTCATGGACGGTGCGGATGGGCTTGCCGGAGGCATGGCGTTGTTCGGCTTCGGC
GGGTATGCGGTCGCGGCGCTCGGCGGCGCACAAGAGTCGCCCGATCTCGTCGTTTCCGGA
GCGGCGGTCGCCGGCGCGGCGCTGGGCTTTCTTCTGCTCAATTTCCACCCGGCGAGACTA
TTCCTTGGCGACGCCGGCTCGATTCCGCTCGGCTTCCTGGCCGGTGCGCTGGGCTACTGG
GGCTGGCGTACCGATGTCTGGCCGATCTGGTTTCCGGCCATGGTGTTCTCGCCCTTTATT
CTCGATGCATCTGTAACACTTCTGAGACGCTTGTTACGCGGAGAAAAGTTCTGGCAGGCG
CATCGCGAGCATTATTATCAGAGGATGGTTCGATCGGGCGTCAGCCACGGTCGGACCGCT
CTTTATTGGTACCTCATCATGCTCGCCGGCATAATTGTCGCCGTGTGGGCAAAGGGCCGC
CCTGAACTGCAGCAATGGCTGTCGTTCCTCGCGTGGTATGGCGTCCTGGCATGGTTCGGA
TGGTTGATCGACATGCGTTGGCGCCGGGTTCAAGTGGCTGCCGAAAACAATTCTTGA
>IST439_01742 NAD-dependent epimerase/dehydratase
GTGAGTGATCTCGTCATCAGCGGCGCGAACGGCTTCGTTGGCCGAGCGGTCTGCCGTCGC

GCGCTGGAGGCCGGGCACACCGTCACGGCGCTGGTCCGGCGACCGGGCGGGTGCGTCGAT
GGCGTACGCGAATGGGTGCATGACGCAGCCGATTTCGACGGCCTTGATGCGCGATGGCCG
GCCGACCTGGCAGCCGATTGCGTGATCCATCTCGCCGCTCGTGTGCATGTGATGCGCGAC
GAGTCGCCCGATCCCGATGCCGCGTTCGACGCTACCAATGTCGCCGGCACGCTGCGTCTC
GCCGAGGCGTCGCGCAATCACGGCGTGCGCCGCTTCGTGTTCGCGAGCAGCATCAAGGCT
GTCGGCGAGGGCGATGGCGGTGTGCCGCTATCGGAAGCCGTCGAACCCGTTCCGCAGGAC
GCGTACGGCCGCACCAAGCTACGCGCGGAGCGGCAACTCGCGCAATTCGGCGCGTCGGCG
GGGCTCGACGTCGTCGTCGTGCGTCCGCCGCTCGTGTACGGCCCGGCCGTCCGTGCCAAC
TTCCTGAGGATGATGGACGCGGTCGCGCGCGGGGTGCCGTTGCCGTTCGGCGCCGTTTCG
GCGCGCCGCAGCGTCGTCTACGTCGACAATCTCGCCGATGCGCTGCTGCGTTGCGCAATC
GATCCACGCGCGGCCGGCGAGTGCTTTCATATCGCCGACGACGACGCCCCGACGGTCGCG
GACCTACTGCGCCTCGTCGGCGACGCGCTTGGCCGCCCGGCGCGGCTAATTGCAGTACCT
CCTGCACTACTGCGGGCGGCCGGGGCGCTGACAGGCCGCCGAGCGGTTATCGACCGCCTG
ACAGGGAGCTTGCAGCTCGATACCGACCGGATAAGGCGCGTACTCGACTGGCATCCGCCT
TATACGACCCGCGAAGGCCTTGAAGCGACCGCCGCGTGGTATCGTTCGCGCGATACACAA
CAATAG
>IST439_01743 glycosyl transferase family protein
GTGCTTGACCGGATGAATGTAGCCTTCGACGTGATTACAGGCCACGGCAACGTCGGCTAT
GGCCGCGGCCATAATCTCGCGATCGAGACGGTATGCAGCCGTTACCACCTCGTGCTGAAT
CCCGACATCGATCTCGCGCCGAATGCGCTTGCGAACGCGCTTGATTTCTCGATGCCCAT
CCAGAGGCCGGCCTGCTGACGCCCTACATTGGCGACGAAGCCGGCCGCATCCAGTATCTG
TGTCGTCGCTATCCCGCGATGCTCGATCTGTTCGTGCGAGGTTTTCTGCCGTCGGTTTTT
CACAAATCCTTCGAGCGCCGGCTGGCTCATTACGAAATGCGTGACGAGATCAACGACGCG
GATATCGTCTGGGATCCACCGATCGTGAGCGGATGCTTTATGCTCTTCCGGACCGACCTG
CTCAAGCGTCTGAATGGTTTCGATCCACGGTATTTTCTGTACTTCGAAGACTACGATCTC
AGCTTGCGGACCCGTGAGCTGGCGCGGATCGCATATGTGCCTGCGGTACGTGTGCTTCAC
CACGGCGGTGGTGCTGCCCGCAAGGGATGGGCCCACATCCGGATGTTCGCCGCGTCGGCC
TTCAAGTTCTACAACCGCTTCGGGTGGAGGCTGTGGTGA
>IST439_01744 hypothetical protein
GTGGCCAACCCCGCCCCGCTCACCTCGGTCGAAGGCGGACTGGCACACGGGAAGATGCAT
GAAACTTGGCGGATGGCGCTCGCCGAGCAGGTGGATTCGATTTGGAGCATGAAAGAGGTG
CGAGAGTGCGAAACGGAAGTCACGAGTCAGGGCAGCACAATGGACGTCTATAACGACACG
CCCCAGGTCGCGAGCGGCAACGGGCGGAATGGTATGGAGGCGATCACGCAGCTCTGTGAT
ACCGAACGAGCGACAGGACCGTTCAGGGATCTGTCGCTTACGCTATATCTATTTACACGC

CGACTTTATGACGCAGCACTGAAGGACGGTGTAAGCGATCTGTTTTTCTTCGCCAGAGAA
GGACTTTTGCTGAAGCAAATGTTCGACCGGGTGCAAGCCAATGAGGACAAGACACCCATA
CGATCTCATTACCTAAAGGTGTCTCGTCGTTCGACCTTTCTACTGTCACTGCGCCCGTTG
GACCAGGAGCAGTTTGAAGTTCTCTTCCGACAGTACAGAAGGATCTCGATCGCCGAGTTT
CTGAAGAGCCTAGCGCTGGAAGAATATCTTCTGACGTTCGCTACCGAGCTTAACGTCGAA
CCGTCGCTGCTGGCCGAGCGCAGTGCAGATTTGCCGCACGATCCACTTTTCGTCCGACTG
CTGGACCTGCCGTCGTTTGCGCAAGTGTACGAGAAGGAGCGACTTGCGCGCAGCAAGGCG
TTCGAAAATTACGTGGGCAGTTTCTGCAACGGCACCATCCCTGCGCGGTTGAGTGTTGTT
GACGTGGGATGGAAGGGGTCAATCCAGGACAACGTATTCGGATGGATGCGTAATCTCCGG
GGAGACGCGGCAAGCGTCAAGGGCTACTACGTCGGCCTCATCGCAACCGGCAATGCCGGC
CCCTCCAACGAGAAGGCAGGATTGTTATTTGAGAGCATCGGCGAGCGCACCGCGGGCTAC
TACGTGTTCAACGAAAACCGGTCACTCTTTGAAGTCTTGCTGCATGCCAACCACGGGTCG
GCACAGCGCTATATTCAGACATCCGCGAACGAGGTCATCGTCGTAGAAGACTCGTTTGAA
GAAGGTCCGATGATCGAAGCGTTTGTTAAACCGGTAGCCGATTGCATCATGGCCTCGTTC
GTTCGTATCGCGGACGCGATGGCTACGCTTGATGTGTCCGAGCAAGACCTTCTGGCCTGC
GCAGTCAAACGCCATTCACGCATGGTGTTCCGGCCTTCATCAAGCGAAATCGAATGGATG
CGCTCACTGTCGCACGTTGAAAATTTCGGTGTCTTCGAGGCGTCGACGTTCGACAATCGC
CGGACCTCGCCTTCGTTGGGTTCCCGGCTAAGATTCTCGATCGATGTATTCAGACACGGT
CAACGCGACCTCGGTTTCTGGCCTTGGCTGACGATTGAAGAGCGCGCCCTCTGGGGAATG
GCAGGTGTCTATCGGATCATTCGCATGTGGCAGAACCAACGGACCATGCGTCTGCACTCG
AGCCCAACGGACAAAAAGGCAGTACCTAAATGA
>IST439_01745 group 1 glycosyl transferase
ATGAAAGTGTGTTTTCTTGTGGGGTCCATGGCGATCAGCGGTGGAACCTACGTCATTGTT
CAGCACGCAAGCTATTTGCGTGACCACGGCTACGATGTCACTCTCGCCGTTCAAGAACCC
TTCACGCCCGAGACGCTGGCGTGGCACAACGAGGCGCCTCGGCTGCGTTGCATTCCCATT
GCGACGGCCCGTTCCGAGAAATACGACCTCGTGATCGCCACATGGTGGAAAACGGCGCTC
GAGCTTCATGAATTCGATGCTGCCCGTTACGCGTACTTTGTCCAGTCGATCGAGTCCCGA
TTCTATCCGGAAGCGGAGATCCCGTTACGCGCGCTCGTCGACGCGACCTACAAGCTCCCA
GTGACTTATGTGACGGAAGCGACGTGGATCAAGGATTACTTGCAAAGCCATTTCGGGCAG
GAAGCGTCACTGGTCAAGAACGGTATCCGGAAGGATGTCTATACCCCGACCGGGCCAGCC
GTCGAAGCAAGGAGTCCGACTCGTCTCCGCGTATTGGTAGAAGGACATTTCGGAGTACCG
TTCAAAAATACCGCGCTGGCAATTCGTCTCGCGAGGGAAGCAGGTGCAAGCGAAATCTGG
CTGCTGACTGGCTCACCCGTGCAATGGATTCCCGGTGTGTCCCGAGTATTTTCCCGAGTA
CCTATGGTCAAGACGGCGGAAATCTATCGGTCCTGTGACGTGCTACTGAAGCTGAGCACG

GTCGAAGGAATGTTCGGACCGCCTCTTGAAATGTTCCACTGTGGCGGAACAGCAGTCGTC
TTCGACGTCACCGGGCACGATGAATACATCGTCGACAACGAAAATTCGCGGGTAGTATCG
ACAAACGATCTGGACGGAGTTGTGAAAACGATTCGGACGCTCCTGAACGACCGAAGCGAA
CTCGCTCGGCTTAAGGCAGGCGCCTTGCGCACAGCTCAAGCATGGCCCCAATGGGAAGAC
GCATCTGCGAAATTCTCAGACTGGATTCGCGATTGCTTGCAAGGGCCGGTCGTGAATCGA
GATGAGTGTGCAGCAGTCATCGCAAAGGCCTGGAGCGACTATGACCGTGACGAAAAGCTG
CGTCTCGCACAAACTCGCCAGTCCGTCAGCCGCATGAGGCTGCGTGCGCTCGCCGGAAAA
TTGCCAGGCTCGTGGACACGACGTCTCAAACAGCTCGAAGCAGTGAGCGAAGTGTTGGTT
GGCAAGCGTAAGGCCTATTGA
>IST439_01746 HAD superfamily hydrolase-like protein
ATGTACAGACTGAAGACCATAGACGTGTGGGACACGCTGCTCCGGCGCGATTGCCACCCG
GAATTTGCAAAACTCGCAACGGCGTTGCACGTGTTTCTCGATCCGTCGATCGGTGTTTCC
TGCCACTATGAAACGGCGTGGGACATCTATTGTCGGAGAGTCGAGCAAGAAATGCTGCTG
GCCGATCGCGCGCGCGCAGCGGGAAAAGACAACGAGTACGAGATCGTCGAGGTACTGCGG
CAATGGCTTACGCAGATCGTCGATCGACCCTACGACGACTCCCTGCCGTCCCGACTCGCC
GATTTCGAACTGGGCTTCGAACTTCGACACACCTATCCTGACCCAACGATCAGGGATGTA
TCCGAGCAGTATCCTGCGGAAACGACGCTGTTCCTGTCGGACTTTTATATGTCCGCGGAT
CGCCTTTCCACGCTGCTGCATCACCACGGAATCGACACGTTTGTGCCGGGAGGCTTGAGT
TCCTGCGACGTCGGTATCAACAAACGCTCGGGCAAGTTTTTCACTACGTCGAAGATCTC
TATCGAGTAATGCCGAATGAGCATGTTCACATCGGCGACAATTTGACGTCGGATCGCGAT
GTGCCTCACGCGCTTGGTATCAATGCAGTCCATTTTCAACCGGAAGCAGAGCATGCGAAG
CGACTGGCTCGCGAATCCGTCTTCGAGGATAGAGGCGAGTTGTTTCGCAAGATTCAGGCA
GAAGTAAAGGTAGCAGCAAGTGGCTACCAATTTGCCCATGAGCAGTCCGCTGAACGCGAA
GCCGCGTTCAGCGCAGGGATTCAGTCGAGCGTGCTGGTGATTGGCTTCTGTCTCTACGTT
GCCGAAAATGTGCTGTCTGGTGGGCTCAAGAGTCTGTACTTCTTTTCACGTGAAGGCGAA
TTCTTTGTACGTGTGTGGAATGCACTATTCCCGAGCGGACAGCATGCCGGTCACACACTT
CCGCACGCCTCTCTACTCGAAGTGAGTCGCCTTTCGACGTTCTGTGCGTCACTGCGGGAG
GTCACCCCGTCAGAGATGCGCCGAATCTGGAATCTATATACAACACATTCTCTCGAAGCA
CTCTTGAAGTCGCTGGGGCTTGAACCTCGCTCGTTCGCGGAACTGGCAGCGAAGCATCAG
TTGCCGTTGACTGAAACGATCCAGCATCCATGGGAAGACGGCCGTGTCAAACGCCTCTTC
GACGATCCGCAGTTTGTTCATCAAATCACGGAGAAGATTCACGCCGATCGTCGTGCATTG
CTAGCTTACTTGGCGACACAAGGGTTACGTGATGATGGATCGGTAGTGAGCGTCGTCGAT
ATTGGTTGGCGCGGCACCATTCAAGATAATCTTGCATGGCTTCTGCCCTCCGTACAATTC
CAGGGGCAATATCTGGGATTGCAAATGTTCCTGAACGAGCAGCCTGCGAATACGCACAAG

CAAGCGTTCGGACCCAATGCAAATGAAACACTGGAACACTGCCATCTTCTCGACGCCGTG
TCTCCGCTCGAGATGTTGTTCAGTTCACCTCTCGGTTCAGTATCCGGCTATCGCATGAAC
GATGACGGCACCGGTGCAGTTGCATTTCGCTCGGAAAGCGATATGGAACACGCTTCCAGC
AAAAATTTCGTCGAAAGCTTCCAGGATGGCGTGGTTGAAGCCGCGAAGGTGTGGGCCCGC
TACGTCGAAACATGTGCGATTGCTGCGTCAGAACTTCGCGATCCGGCGTTGGCAACCTGG
ACCAGGTTGATCGAACGTCCACACGAAACCTTGGTCGACGCATACTCGGCACTCAATCAG
AATGACGTGTTCGGTGCCGGTCAGTTCGTCAGCAAACGTGCCACCCCGGGAGTAGGCAAG
TTGTTGTGGGGAATCGTTTCGCGCGATGCGCGTCGGGAAGTGATCATGTACGTTCGTCAA
ACCCAATGGACCGCCGGAATCTGGGCGCGTCGCGACTTGACATTGGGAAATCGGCTGGCG
CTAGTGATGGTGTTGCGATTGGCCAAAGGCTACAAGCGATTTATGCAGCGAAGAACCGCC
CAAAATCGCACCCACAGTCGTCTAGCCAAGGAGCGGCCAGGGTCGTCGGACGGTCGTACA
TGA
>IST439_01748 type 11 methyltransferase
ATGGAACGCATGCATTCCGATCATTTCCAGATGCAGCACCTCGTATATGAGCATACGCAT
CGCTATCAATTGCTTTCGCGTATCGCTTACGGGCATGTCGTCGACTGCGCATGCGGTATC
GGTTACGCATCTGAAATTATGGCGGCTGCCGGGGTCCGGTCCTACCACGGTTTCGATATT
GATGAGCAGTCGGTTCAACTGGCGGTCCAACGATTTGCGGCTGACGGACGTCAGTTTACG
GTCGGCTCGATTCTGTCGCTGCCGCTTGAGGATGCGTCGGTCGATACTTTCTTGTCGCTG
GAAACACTTGAACATTTGCGGGAGCCTCAGCGCGCGATTCACGAGATTACCCGTGTGCTG
AAGGCGGACGGGATCTTCATGGGCTCGGTGCCGACGCGTTCTTACGATGAGCGTTGTCAC
GAGGTTTACGGCCCGAACCCCTATCATGTGACGCGGTTCGACGCAGACGAGATCGTTCAA
CTGCTGCGAACCGGGTTTGAGTACGTATGGCTTGGGGTCATCAGCCTCGACGTAGTGTCG
ATCGTCCGGCCTGTGCCGGCCGCGAATGCAGGTCCAGCGGAATGGGTCGGCAACAGCAAG
TGGGACGCGACAAAGTATGGTTCGTTCATCTTCGTCGCGTCACATCAGCCTGTTGACGAA
CTGGTCCGCAAGCTGCTTCCGCAGCAATGCGCGGTGCTGCATGGGTTGCCTACTGTTGAA
GTGGACGCAGAGCAGACTATGCCGCTCTACAAGACAATCACGGAAATCGAGGGTCTTGTG
AGGGATCGCGACGCCGCATTGGATCGATCGGAAGGAATGATCCGGGACCGCGACGCTGCG
TTGCACCGGATGGAAGAGATGATTCGAGACCGCGACGCGGCAATTCGAAGCAATGAGCAG
ATGATCAATGACCGTGACGCATTGATCGCGCAACTCGAACGAATGGTCGCTGACCGGGAC
GCGGCGATCGCATCGAATGAGAAGTTGATCAACGAGCGCGACGCGTTGCTGCGAGCTAAA
CGCACAGACTGA
>IST439_01749 ABC transporter-like protein
ATGTCCTCTAATGCGGTCATTTCGGTTACCAGTCTTGGCAAGTGCTATCAGCTGTACGCG
AAGCCGAGCGATCGCCTCAAGCAGATGCTCTTCGGGCGGTTTAAGCGCTATTACACGGAG

TTTTGGGCGCTGAGAGACGTGTCGTTTGACGTCCGTAAAGGTGAAACGATCGGCATCATC
GGGCGGAATGGCGCCGGTAAGTCGACGCTCTTGCAGATGTTGTCAGGCGTGCTCCAACAA
TCGACCGGGCAAAGCGAGGTGCGTGGTCGCGTGTGTGCGCTGCTGGAACTCGGCGCCGGT
TTCAACCCTGAATTCACCGGGCGCGAGAACGCGATTCTGACCGGCGCAATTTATGGTATT
CCTGGCGAGCAAATGGAAGCGAAGCTCCGGGAAATCGAAGCGTTCGCGGATATCGGTGAT
CATATCGATCAGCCGGTCAAACATTATTCGAGCGGTATGTATGCGCGTCTTGCGTTCTCG
GTCGCGATCCACGTTGATCCCGAGATTCTGATCGTCGACGAGATACTTGCGGTGGGCGAT
GCATTGTTCCAGCGCAAGTGCATGAACAAGTTCTACGAATTTCGCGATCGTGGCTGCACG
GTGCTCTTCGTGACTCATGATGCGTACCAGATCAAGAGTACGTGTCAACGAGCGTTGTAC
TTGAAGAACGGCCGACAGGTCGCGTTCGGCGATGCCACGGAAGTCGTGGATCGTTATCTG
CAGGATCTCGAACTCATTGAGAGTAAGCCGGCTGATGTGGTACAACGGGGTGCGATCGCT
GACGATGCGCCGCAGTCCACGGCTGTAGCGCAGCCGGCAAACCTGATTCGGATCACGGAC
GTGACGCTGACCGACGAATTTGGCTCGCCCATCACGCAGGTGCGAAGCGGTCAGACAATC
GACATTCGTTTCCACTATCACGCGCCGCACAAACAGTCGATTCCGAAGGTATCGTTTGTC
GTCAATCTCTATCGCCACGATTCGTTTTACATCTGTGGCACGACGACGATCATGGATGGT
CTGGAGCCGTTCGCAACGGAGGATGAGGGCGAGGTGATTGTGCGCTTCCCTGATCTCCAA
CTTCTTGCGGGACGTTATATGTGGCGCGTCGCGATCAACGACGAGCGAGGGCTTGGTATC
CTGGCAGAGGCAACGCCGGTCTGCGCGTTTCAGGTGGTCGACGATTTCCAGGCGGTTGGT
CTCGTCAACGTGCCGCGGCAGTGGATCGTACACGGCCAGGAAGTAGAAAAGAGAGAACTT
ATTCCAAAGGTTTGA
>IST439_01750 ABC-2 type transporter
ATGAGGATAATTTCTCCCATCCTCCGGGGTGTCCGTTCCATCGTGTCTCATCGTGACTTG
CTCCGCCGCCTCGTGTGGCGGGACATCCAGGGGCGCTATCGTGGATCTGCAGGTGGGCTC
GGCTGGGCGATCGTTACGCCGCTCGTGCTGTTGAGCGTTTATACGTTCGTCTTCAGCTTC
GTGCTCAAGTCACGCTGGATCGGCGTGCCGAATCAGGATTCGCACGCTACGTACGCGATC
ATGTGTTTTTCGGGGCTCATTGTGTACAGCGTATTTACAGAATGTATTTCACGCGCGCCG
ACGTTGATGACCGGCAACGTGAACTACGTGAAGAAGGTCGTGTTTCCGCTGGAGATTCTT
CCAATCGTGTCGCTCGGAAGTGCGCTGTTCCATTTCGCAATCGGCTTCACCGTGCTCCTG
GTATTCATCGTGGTGTTCGGCTCGGGCCTGCCACCGACCGCTTGGGCGTTCTGGCTCGTG
CTGCCGCCGCTCATGCTCTGGTGCATCGGTCTTGGATGGATCCTTTCGTCACTCGGCGTG
TTTCTGCGTGACCTGGGGCAACTCGTTACACTGGTGCTGAGTGTGTTGATGTTCGCTACG
CCAATTTTCTATTCCGCCGATAGCCTGCCGGAGCAATATCGCATCTACATGGACCTCAAC
CCGCTATCGTTTGTGATCGGGACATTGCGAGAAACCGTCATCCTGGGTCGATATCCCGAT
CCGTCGTCGTATTGTCAGCATCTTCTTCCGGGCATCCTGGTGGCAGTTTTTGGCATGTGG

TGGTTCGAACGTACGCGTGGAGGGTTCGCCGATGTCCTCTAA
>IST439_01751 dTDP-4-dehydrorhamnose reductase
ATGAGTGATTGCGGTGTGAGCGTACCGGAACAGCGGACGATTCTGCTTACCGGAGCGAAC
GGGCAAGTGGGGTTTGAACTTGCGCGAAGCCTGCAGGGATTGGGGCGCGTCATTGCCTGT
GATCGCCGGCAGCTTGACCTGGCTGATCTTGACGGCATTCGCGACGCCATGCGGGCACTG
CGTCCGGCACTGGTCGTCAACGCGGCTGCGTACACAGCCGTCGCCGATGCAGAGCGGGAC
GTGACCGGTAGCATGCGGATCAATGCCGACGCGCCGCGGGTGCTGGCGGAAGAGGCGAAG
CGTGTCGGTGCGGCGCTGGTCCATTATTCGACCGATTACGTATTTGACGGGCGAAAGATG
GGCGCGTACGTCGAGGACGATGCGCCGGCCCCGCTGAACGCATACGGCCGTAGCAAGCTC
GCAGGCGAGGAGGCGATTCGGGGGACTGGCTGCGACCACCTGACTTTCCGTACGAGTTGG
GTGTATGACCTGAGGTCCAACAATTTCTTGACGACGATGCTGCGCATCGGGGCCGAGCGT
CCCGAGTTGCGTGTGGTCAGCGATCAGTTTGGCGCGCCGACGTGGTCTTACGCGATCGCC
AGCCTCACGGCGCAGGTGCTTGCTCAGGCGATCGCAGCCGGCCGCATGCCGGAGCGATGG
TCAGAGTGGTGGTCCACGCATACTGGTGTGTACCATCTGACCGCAATGGGCGAGACATCT
TGGCACGGCTTCGCGGAAGCGATATTTGCTGCGTCCAATTGCACCAACAAGCCTTCGGTT
ATTCCAATCGACGCTGCGTCGTACCCGGGTCCCGTGCTCCGGCCGGCCAACTCGCTGCTG
TCGAACGATAAGCTCGCATGTGCGTTTGGTTTACGTGCGCCGGACTGGCGCTTTTTGCTG
AACCTTTGCCTGGCCGACGCGCACTCGAGTCGCGATGCCGAACGGAACCGGTCGATTGCC
TAA
>IST439_01752 dTDP-4-dehydrorhamnose 3,5-epimerase
ATGGCCATCCAGGTAACAGCAACGGCATTGCCGGAAGTCAAGATCATCGAGCCAAAGGTA
TTCGGCGACGCGCGCGGCTACTTCTACGAGAGCTTCAATGCCCAGGAGTTCGCCGAACAG
GTCGCGCCGGGCGTCGAGTTCGTACAGGACAATCACTCGCGCTCGATGCGCGGCGTGCTG
CGCGGGCTGCACTACCAGATTCAGCATGCGCAGGGAAAGCTGGTGCGCGTGGTCGAGGGC
GAGGTGTTCGATGTCGCGGTCGATGTACGTCGCAGCTCGCCGAACTTCGGCAAATGGGTC
GGCGTCCGTCTGTCGGCCCGCGATCATCGGCAGCTATGGGTCCCGCCGGGCTTCGCGCAC
GGGTTCGTTGTGCTGTCTGACATTGCCCAGTTTCTGTACAAGACAAGCGACTATTGGTTT
CCGCAGCACGAACGGAGCCTGCTGTGGAATGATCCGGCGATCGGGATCGATTGGCAGATA
GACACGTTGCCGACCCTGGCAGCCAAGGATGCGGCCGGGAAGCGGTTGTCCGAGGCGGAA
TGTTATGAGTGA
>IST439_01753 glucose-1-phosphate thymidylyltransferase
ATGGCACGTAAAGGCATTATCCTTGCCGGCGGTTCGGGCACACGGCTGTATCCGATTACG
CATGTCGTATCGAAGCAGCTGCTGCCGGTGTACGACAAACCGATGATTTACTACCCGCTG
TCGACGCTGATGGTGGCGGGCATCCGGGACGTGCTGATCATCTCGACGCCGCACGACACG

CCGCGCTTCGGAGCGATGCTGGGCGACGGCAGTCAGTGGGGCATGAATATCCAGTTCGCG
GTGCAGCCGTCGCCGGACGGGCTCGCGCAGGCCTTCATCATCGGGAAGTCGTTCATCGGC
AACGAGCCGTCGGCCCTGATTCTCGGCGACAACATCTTCTACGGACACGACCTCGCGAAG
CAACTCGAACGGGCCGATGCGAATACGAATGGCGCAACGGTGTTCGCGTATCACGTTCAG
GATCCCGAGCGGTACGGCGTGGTCGAATTCGACCGGGATTTCCGTGCGTTGTCGATCGAG
GAAAAGCCGGTCAAGCCGCGTTCGCACTACGCGGTGACGGGCCTGTACTTCTACGACAAC
CGCGTGTGCGATATCGCGTCCGACATCAAGCCGTCGCCGCGCGGCGAGCTCGAGATCACC
GACGTGAACTCGCGCTATCTGGCCGACGGTGCACTCGATGTCGAGATCATGGGGCGGGGC
TATGCGTGGCTCGATACGGGTACGCATGATTCGCTGATCGATGCGGCAATGTTCATCGCG
ACGCTGCAGAAGCGCCAGGGGCTCGTCGTGGCGTGCCCGGAAGAGATCGCCTACCGCAAG
CGGTGGATCGACGCCGAGCAGTTGGGCAAGCTCGCCGCACCGCTGTCGAAAAACAGTTAC
GGGCGATATCTCCAACATATTCTTTCTGACCAAGTCGCATGGCCATCCAGGTAA
>IST439_01754 dTDP-glucose 4,6-dehydratase
ATGATCCTGGTTACCGGCGGCGCGGGTTTTATCGGTGCCAATTTCGTACTCGACTGGATG
GATGCTTCCGGCGAGGCCGTGCTCAATGTCGACAAGCTGACCTATGCGGGCAACCTGCGG
ACGCTGGTCCCGCTGGACGGGAATCCGGCCCATGTGTTCGCCCGTGTCGACATCTGCGAT
CGCGCGGCACTCGATGCGCTGTTTGCCGAGCACAAGCCGCGCGCCGTGATCCACTTCGCC
GCCGAAAGCCATGTCGACCGTTCGATCCACGGACCGGCCGATTTCGTGCAGACTAACGTC
GTCGGTACGTTTACGCTGCTCGAAGCGGCACGTACGCACTGGAATAGCCTGAACGATACG
GACAAGGCGGCCTTCCGCTTCCTGCACGTGTCGACCGACGAGGTGTTCGGATCGCTGTCG
GCCACCGACCCGCAATTCTGCGAGACGACTCCGTACGCGCCGAACAGCCCGTATTCGGCG
ACGAAGGCCGGCTCTGATCATCTGGTGCGTGCGTATCACCATACGTACGGCCTGCCGACT
CTCACGACGAATTGCTCGAACAACTACGGCCCGTACCAGTTCCCCGAGAAACTGATTCCG
CTGATGATCGCGAACGCGCTCGCGGGCAAGCCGCTGCCGGTCTACGGCGACGGCCAGAAC
GTGCGCGACTGGCTGTACGTCGGCGACCACTGCAGCGCGATCCGCGAAGTGCTCGCGCGC
GGCGTGCCGGGCGAGACGTACAACATCGGTGGCTGGAACGAGAAGAAGAATCTCGACGTC
GTGCATACGCTGTGCGATCTGCTCGACGCGGCGCGGCCGAAGGCGGCCGGTTCGTACCGC
GAGCAGATCACCTACGTGAAGGACCGTCCGGGCCACGACCGCCGCTACGCGATCGATGCG
CGCAAGCTCGAGCGCGAACTCGGCTGGAAGCCCGCCGAGACGTTCGAGACGGGGCTCGCG
AAAACCGTGCGCTGGTATCTCGACAATCAGGCGTGGGTGGACGACGTCGCGTCGGGCGAT
TATCGCAAGTGGGTCGAAACCAATTACGCGCAACGCACGTGA
