# Supplementary material for: Structure of O-Antigen and Hybrid Biosynthetic Locus in Burkholderia cenocepacia Clonal Variants Recovered from a Cystic Fibrosis Patient
Source: Front Microbiol. 2017 Jun 8;8:1027. doi: 10.3389/fmicb.2017.01027 (PMC5462993; doi:10.3389/fmicb.2017.01027)
Supplement: Supplementary file 2 [file Table_2.pdf]

Supplementary Material

Structure of O-antigen and hybrid biosynthetic locus in *Burkholderia cenocepacia* clonal variants recovered from a cystic fibrosis patient

A. Amir Hassan[1§], Rita F. Maldonado[1§], Sandra C. dos Santos[1§], Flaviana Di Lorenzo[2§], Alba Silipo[2], Carla P. Coutinho[1], Vaughn S. Cooper[4], Antonio Molinaro[2], Miguel Valvano[3] and Isabel Sá-Correia[1*]

*** Correspondence:** Professor Isabel Sá-Correia: isacorreia@tecnico.ulisboa.pt

[§] These authors contributed equally to this work

1 Supplementary Tables:

Table S2: Primer sequences used to amplify the desired regions with detailed description

Primers	Primer Sequences	Description
primer 824	5'-GCCCATTTTCCTGTCAGTAACGAGA-3'	Screening of the transformants
pSC rev	5'-GATGCCTGGCAGTTCCCTACTCTCG-3'	
WbiI–flag–NdeI	5'-GCTACATATGGACTACAAGGACGACGACGACAAGTTGCAATCCAGAGCATCTTGGCT-3'	Cloning of *wbiI* with an N-terminal FLAG epitope incorporated to the expressed protein
WbiI_439_XbaI	5'-TAGCATCTAGATCAGCGGTTCGATGCGACTCTCAC-3'	
wbiI 439 NdeI	5'-ACGCTCATATGTTGCAATCCAGAGCATCTTGGCTG-3'	Cloning of *wbiI* with a C-terminal FLAG epitope incorporated to the expressed protein
WbiI–flag–XbaI	5'-CGTTCTAGATCACTTGTCGTCGTCGTCGTCCTTGTAGTCGCGGTTCGATGCGACTCTCAC-3'	
Bmul_2510-439-NdeI	5'-AAGGCCTACATATGTACAGACTGAAGACCA-3'	Cloning of *bmul_2510* with a C-terminal FLAG epitope incorporated to the expressed protein
Bmul_2510-flag-XbaI	5'-CGTTCTAGATCACTTGTCGTCGTCGTCGTCCTTGTAGTCTGTACGACCGTCCGACGACCC-3'	
Primer P1	5'-TGAAATTCAGCAGGATCACAACGCTCATATGTTGCAATC-3'	Cloning of both *wbiI* and *bmul_2510* with a C-terminal FLAG epitope incorporaated to the expressed protein using the Gibson assembly method
Primer P2	5'-GCGACCTCCTCGTTCTAGATCACTTGTCG-3'	
Primer P3	5'-ATCTAGAACGAGGAGGTCGCGCTCATATGTACAGACTGAAGACC-3'	
Primer P3	5'-GATCCCCGGGTACCATGGCACGTTCTAGATCACTTGTCGTC-3'	
