# Supplementary material for: Structure of O-Antigen and Hybrid Biosynthetic Locus in Burkholderia cenocepacia Clonal Variants Recovered from a Cystic Fibrosis Patient
Source: Front Microbiol. 2017 Jun 8;8:1027. doi: 10.3389/fmicb.2017.01027 (PMC5462993; doi:10.3389/fmicb.2017.01027)
Supplement: Supplementary file 3 [file Table_3.pdf]

Supplementary Material

Structure of O-antigen and hybrid biosynthetic locus in *Burkholderia cenocepacia* clonal variants recovered from a cystic fibrosis patient

A. Amir Hassan[1§], Rita F. Maldonado[1§], Sandra C. dos Santos[1§], Flaviana Di Lorenzo[2§], Alba Silipo[2], Carla P. Coutinho[1], Vaughn S. Cooper[4], Antonio Molinaro[2], Miguel Valvano[3] and Isabel Sá-Correia[1*]

*** Correspondence:** Professor Isabel Sá-Correia: isacorreia@tecnico.ulisboa.pt

[§] These authors contributed equally to this work

1 Supplementary Tables:

Table S3: Monosaccharide compositional analysis of the LPS/LOS isolated from *B. cenocepacia* isolates IST4113, IST4129, IST4134 and IST439.

Strains	Monosaccharide composition
**IST4113, IST4129, IST4134**	4-amino-4-deoxy-L-arabinose (L-Ara4N)
	D-glucose (D-Glc)
	D-galactose (D-Gal)
	D-glucosamine (D-GlcN)
	L-*glycero*-D-*manno*-heptose (L,D-Hep)
	3-deoxy-D-*manno*-oct-2-ulopyranosonic acid (D-Kdo)
	D-*glycero*-D-*talo*-oct-2-ulopyranosonic acid (D-Ko)
**IST439**	4-amino-4-deoxy-L-arabinose (L-Ara4N)
	D-glucose (D-Glc)
	D-galactose (D-Gal)
	L-*glycero*-D-*manno*-heptose (L,D-Hep)
	3-deoxy-D-*manno*-oct-2-ulopyranosonic acid (D-Kdo)
	D-*glycero*-D-*talo*-oct-2-ulopyranosonic acid (D-Ko)
	4-amino-4-deoxy-L-arabinose (L-Ara4N)
	D-glucose (D-Glc)
	D-glucosamine (D-GlcN)
	D-ribose (D-Rib)
	D-galactosamine (D-GalN)
