# Supplementary material for: Structure of O-Antigen and Hybrid Biosynthetic Locus in Burkholderia cenocepacia Clonal Variants Recovered from a Cystic Fibrosis Patient
Source: Front Microbiol. 2017 Jun 8;8:1027. doi: 10.3389/fmicb.2017.01027 (PMC5462993; doi:10.3389/fmicb.2017.01027)
Supplement: Supplementary file 4 [file Image_1.pdf]

*Supplementary Material*

# Structure of O-antigen and hybrid biosynthetic locus in *Burkholderia cenocepacia* clonal variants recovered from a cystic fibrosis patient

**A. Amir Hassan[1§], Rita F. Maldonado[1§], Sandra C. dos Santos[1§], Flaviana Di Lorenzo[2§], Alba Silipo[2], Carla P. Coutinho[1], Vaughn S. Cooper[4], Antonio Molinaro[2], Miguel Valvano[3] and Isabel Sá-Correia[1*]**

*** Correspondence:** Professor Isabel Sá-Correia: isacorreia@tecnico.ulisboa.pt

[§] These authors contributed equally to this work

**Supplementary Figures:**

**Figure S1**

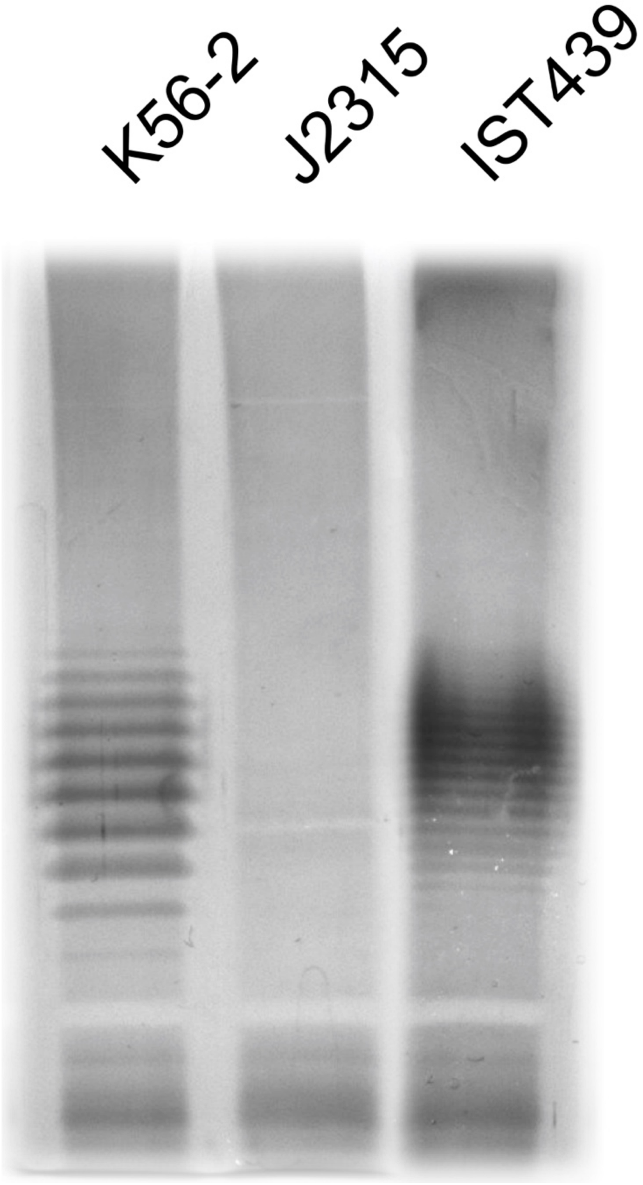


**Figure S1:** Electrophoretic profiles of the LPS extracted from *Burkholderia cenocepacia* IST439 isolate and the (*recA* lineage IIIA) ET-12 reference strains K56-2 and J2315. LPS loading was standardized based on culture optical density. Samples were run in 14% polyacrylamide gels in a Tricine-SDS system and developed by silver staining.
