# Supplementary material for: Structure of O-Antigen and Hybrid Biosynthetic Locus in Burkholderia cenocepacia Clonal Variants Recovered from a Cystic Fibrosis Patient
Source: Front Microbiol. 2017 Jun 8;8:1027. doi: 10.3389/fmicb.2017.01027 (PMC5462993; doi:10.3389/fmicb.2017.01027)
Supplement: Supplementary file 6 [file Image_3.pdf]

Supplementary Material

Structure of O-antigen and hybrid biosynthetic locus in *Burkholderia cenocepacia* clonal variants recovered from a cystic fibrosis patient

A. Amir Hassan[1§], Rita F. Maldonado[1§], Sandra C. dos Santos[1§], Flaviana Di Lorenzo[2§], Alba Silipo[2], Carla P. Coutinho[1], Vaughn S. Cooper[4], Antonio Molinaro[2], Miguel Valvano[3] and Isabel Sá-Correia[1*]

* **Correspondence:** Professor Isabel Sá-Correia: isacorreia@tecnico.ulisboa.pt

§ These authors contributed equally to this work

Supplementary Figures:

Figure S3

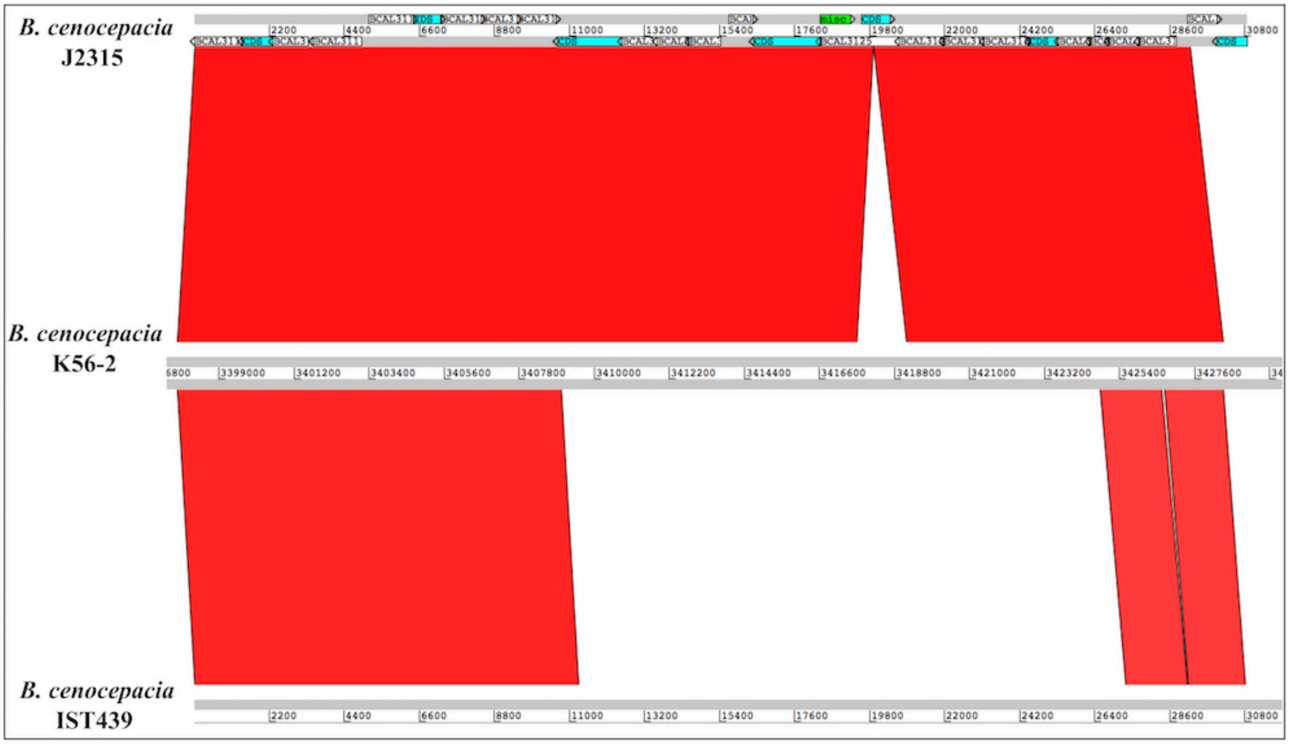

Figure S3: Comparative genomic of the OAg cluster is visualized by Artemis/ACT among *B. cenocepacia* IST439 and the reference strains J2315 and k56-2.
